# KRAS silencing impacts chromatin organization and transcriptional activity in colorectal cancer cells

**DOI:** 10.21203/rs.3.rs-3752760/v2

**Published:** 2024-02-14

**Authors:** Flávia Martins, Ana Luísa Machado, Andreia Ribeiro, Susana Mendonça Oliveira, Joana Carvalho, Rune Matthiesen, Vadim Backman, Sérgia Velho

**Affiliations:** i3S - Institute for Research and Innovation in Health; i3S - Institute for Research and Innovation in Health; i3S - Institute for Research and Innovation in Health; i3S - Institute for Research and Innovation in Health; i3S - Institute for Research and Innovation in Health; Universidade Nova de Lisboa; Northwestern University; i3S - Institute for Research and Innovation in Health

**Keywords:** KRAS inhibition, drug tolerance, therapy resistance, chromatin

## Abstract

Preclinical and clinical data have highlighted the challenges in targeting KRAS mutant tumors, revealing that cancer cells initially sensitive to treatment circumvent KRAS dependence and become tolerant. However, the exact mechanisms governing the transition from a drug-sensitive to a drug-tolerant state remain unclear. Herein, we used 3D culture models of mutant KRAS colorectal cancer cells with distinct KRAS dependencies to show that sensitive and resistant cells undergo distinct chromatin and transcriptional adaptations upon acute KRAS loss. KRAS-silenced sensitive cells presented G0/G1 cell cycle arrest and exhibited a transcriptional signature characteristic of quiescent cells. Moreover, proteomic profiling underscored common biological processes up-regulated in sensitive cells, including nucleosome assembly, gene expression regulation, and mRNA splicing. A detailed analysis of chromatin features revealed that sensitive cells altered eu/heterochromatin states and gained TADs after KRAS silencing whereas the eu/heterochromatin states of resistant cells remained unchanged, and the number of TADs decreased. Strikingly, only KRAS-silenced sensitive cells displayed alterations in how chromatin organizes into packing domains, displaying a lower domain packing scaling. Chromatin packing scaling regulates the interaction between macromolecular complexes and DNA, thus controlling transcriptional malleability and plasticity. Accordingly, although KRAS-silenced sensitive and resistant cells displayed a transcriptomic profile distinct from their controls, only sensitive cells presented higher variability over time, thus suggesting higher transcriptional plasticity. Overall, our findings provide compelling evidence supporting chromatin 3D reorganization and transcriptional variability in KRAS-silenced sensitive cells. This epigenetic mechanism is likely to underlie the remarkable ability of cancer cells to adapt, persist, and sustain malignancy without oncogenic KRAS.

## Introduction

KRAS stands out as the most mutated oncogene in cancer and occupies a pivotal position among the group of key cancer targets (1). Mutations that render KRAS constitutively active occur in numerous cancer types at variable frequencies and prevail in lung (25%), colorectal (40%), and pancreatic (95%) cancers (2,3).

In colorectal cancer (CRC), mutant KRAS occurs at early stages of tumor development, and its oncogenic activity is needed for the progression from a premalignant to a malignant stage and for the development of metastasis (4–6). Oncogenic KRAS signaling fuels cancer cells through strong mitogenic, survival, stemness, invasion, and prometastatic signals (5,7,8) and is also a major regulator of cancer cell metabolism (9). KRAS mutations not only predict an unfavorable response to chemotherapy treatments such as folinic acid, fluorouracil, and oxaliplatin (FOLFOX) (10) but also serve as biomarkers indicating resistance to epidermal growth factor receptor (EGFR)-targeted therapies (11). Mutant KRAS not only regulates crucial cancer activities but also extends its oncogenic signaling beyond cancer cells. It orchestrates a pro-tumorigenic crosstalk with fibroblasts, immune cells, and endothelial cells within the tumor microenvironment, thus expanding its repertoire of pivotal roles in cancer progression. (12–14).

Based on the premise that the inhibition of such a multifaceted, central cancer driver would induce long-term anticancer responses, there have been numerous attempts to develop KRAS-targeted therapies (15). This long-lasting endeavor is becoming fruitful, as the undruggable KRAS has recently become druggable. The first mutant-specific KRAS-targeted therapies were approved for the treatment of non-small cell lung cancer patients with G12C mutations (16), and many other inhibitors for other mutations or the total protein are under development and/or in clinical trials (17–20). Nevertheless, preclinical and clinical data have revealed that targeting KRAS mutant tumors is more challenging than initially expected, as cancer cells can rapidly bypass the dependence on this oncogene, thus recurring shortly after treatment initiation (21–23). In CRC, stable disease occurs in most treated patients, suggesting that cancer cells, while initially sensitive to treatment, tolerate KRAS inhibition and maintain viability. Growth arrest lasts for approximately four to five months before disease progression occurs due to the emergence of resistance (22,24).

However, before resistance eventually develops, a subset of cells must persist in a drug-tolerant state during treatment (25,26). Therefore, there is a compelling need to elucidate the mechanisms by which mutant KRAS cells withstand KRAS loss, as this understanding will offer valuable insights for the development of therapeutic strategies to improve the initial efficacy of KRAS inhibition. Specifically, targeting the mechanisms that CRC cells exploit to transition from a sensitive to a tolerant state could disrupt their intrinsic or adaptive resistance, thereby improving therapeutic outcomes. Expanding on this rationale, our study compels evidence for a non-genetic mechanism that entails chromatin conformational alterations and heightened transcriptional variability involved in the response of CRC cells to the absence of KRAS.

## Materials and Methods

### Culture conditions and siRNA transfection

The CRC cell lines HCT116 (RRID:CVCL_0291) and SW480 (RRID:CVCL_0546) were purchased from the American Type Culture Collection (ATCC). LS174T cells were kindly provided by Dr. Ragnhild A. Lothe (Oslo University Hospital, Norway).

HCT116 and SW480 cell lines were cultured in RPMI 1640 medium (Gibco, Thermo Fisher Scientific, USA) supplemented with 10% fetal bovine serum (FBS; Hyclone, USA) and 1% penicillin–streptomyci (PS; Gibco, Thermo Fisher Scientific, USA). LS174T cell line was cultured in DMEM medium with 10% FBS and 1% PS. All cells were maintained at 37 °C in a humidified atmosphere with 5% CO_2_.

Prior to three-dimensional (3D) cultures, CRC cell lines were seeded in two-dimensional (2D) conditions in six-well plates, cultured for approximately 16 hours, and then transfected using Lipofectamine RNAiMAX (Invitrogen, Thermo Fisher Scientific, USA) in reduced-serum Opti-MEM medium (Gibco, Thermo Fisher Scientific, USA) according to the manufacturer’s guidelines. Gene silencing was performed using ON-TARGETplus SMARTpool small interfering RNA specific for KRAS (siKRAS; L-005069-00-0010) from Dharmacon (USA) at a final concentration of 10 nM. A non-targeting (siControl) siRNA (D-001810-01-50; ON-TARGETplus Non-targeting siRNA #1) was used as a negative control at the same concentration as the siRNA targeting KRAS. Twenty-four hours post-transfection, cells were harvested with 0.05% Trypsin-EDTA (Gibco, Thermo Fisher Scientific, USA) for 5 min at 37°C, resuspended in completed medium, and seeded in 3D CoSeedis (abc biopply, Switzerland) or molds prepared with micro-molds (3D Petri Dish^®^, by MicroTissues, Inc., USA). Cell seeding was performed at a density of 1 × 10^5^ cells mL^−1^, corresponding to 1000 cells per microwell. 3D cultures were kept for 48 hours, and optical microscopy was used to follow cell growth and spheroid formation. KRAS silencing efficiency was monitored by Western blotting (Figure S1).

### Cell cycle evaluation

Cell cycle was assessed using Click-iT Plus EdU Alexa Fluor 647 Flow Cytometry Assay kit (Invitrogen, ThermoFisher Scientific, USA). Briefly, 72 hours after transfection, siControl and siKRAS cells cultured in 3D were gently dissociated with TryPLE^™^ Express (Gibco, Thermo Fisher Scientific, USA), and 5 × 10^5^ cells from each condition were incubated with EdU (5-ethynyl-2′-deoxyuridine) at a final concentration of 10μM, for 1 hour. Cells were fixed using the Click-iT fixative for 15 minutes at room temperature (RT), protected from light. After one wash in 1% bovine serum albumin (BSA) in PBS, cells were resuspended in 1x Click-iT saponin-based permeabilization and wash reagent (washing buffer) and incubated for 10 minutes at RT, in the dark. The Click-iT Plus reaction cocktail was prepared as indicated by the manufacturer to a final volume of 200 μL per sample and incubated for 30 minutes at RT in the dark. Finally, cells were washed once with Click-iT washing buffer and once in PBS. Pelleted cells were resuspended in PBS with RNase A (final concentration of 100 μg mL^−1^) (Life Technologies, Thermo Fisher Scientific, USA) and incubated for 15 minutes at 37°C. Following the last incubation, 1% BSA was added to each sample. Before acquisition in a FACSCanto II cytometer (BD Biosciences), 0.5 μg mL^−1^ of propidium iodide (PI, BD Biosciences, Germany) was added to each sample. Results were analyzed in FlowJo software version X and are represented by the mean ± standard deviation of three biological replicates of each condition.

### Cell viability analysis

Cell viability of siControl and siKRAS cells cultured in 3D was measured using annexin V-FITC and PI double staining followed by flow cytometry. Briefly, spheroids were removed from microwells by flipping the matrices upside-down, followed by centrifugation at 1200 rpm for 5 minutes. Then, the media containing the spheroids was submitted to centrifugation and the spheroid pellet was dissociated for 30 minutes at 37°C with TryPLE^™^ Express (Gibco, Thermo Fisher Scientific, USA). SiControl and siKRAS cells (2×10^5^) were washed with 1x PBS, centrifuged at 300 g for 10 minutes at RT, resuspended in 1x Annexin-binding buffer, and stained with annexin V-FITC (ImmunoTools, Germany) for 15 minutes at RT in the dark. After incubation, 1x Annexin-binding buffer was added and mixed gently. Cells were stained with 0.5 μg mL^−1^ of PI (BD Biosciences, Germany), washed, and then analyzed in a BD Accuri C6 flow cytometer. Results were analyzed in FlowJo software version X and are represented by the mean ± standard deviation of three biological replicates of each condition.

### Spheroid characterization

After 48 hours of culture in 3D, spheroids were imaged under an automated widefield microscope (IN Cell Analyzer 2000). Spheroids’ area, diameter, circularity, and solidity were quantified using Fiji software.

### Cell lysis and protein extraction

Cells were washed twice with PBS, followed by slight dissociation with Accutase (GRiSP, Portugal) for 30 minutes at 37°C. Spheroids were recovered from the microwells by flipping the matrices upside-down, followed by centrifugation at 300 × g for 1 minute. Cells were pelleted and then resuspended in cell lysis buffer containing 1% IGEPAL CA-630, 1% Triton X-100 supplemented with a protease inhibitor cocktail (Roche, Switzerland) and a phosphatase inhibitor cocktail (Sigma‒Aldrich, USA). Cell lysates were further incubated on ice for 30 minutes and centrifuged for 20 minutes (13300 rpm, at 4°C) to pellet the insoluble material. Protein concentration was determined using a DCProtein assay kit from BioRad (USA). The extracted proteins were stored at −20 °C until further analysis.

### Sample processing for proteomics

A hundred μg of proteins from each sample was used for proteomic analysis. Proteins were eluted with an elution buffer (60 mM Tris–HCl, 10% glycerol, 2% SDS, and 5% 2-mercaptoethanol, pH 6.8) at 95°C for 5 minutes. To prepare the samples for proteomic analysis, a single-step reduction and alkylation with tris-2(-carboxyethyl)-phosphine (TCEP)/ chloroacetamide (CAA) was performed in combination with the single-pot solid-phase-enhanced sample preparation protocol as previously described (27,28). Briefly, to reduce disulfide bonds and alkylate cysteines, sodium deoxycholate (SDC) 2x buffer (200 mM Tris pH 8.5, SDC 2%, 20mM TCEP, 80mM CAA) was added to the protein sample and incubated for 10 minutes at 95°C with 1000 rpm mixing. Next, 100 μL of Sera-Mag Magnetic Beads (10 μg μL^−1^, GE Healthcare 45152105050250 and 65152105050250) and ethanol (EtOH) 100% were added to the protein solution (50% EtOH, final concentration), resuspended, and incubated at 22°C for 10 minutes at 1000 rpm. After complete binding, the samples were washed with 80% EtOH. Then, 50 μL of triethylammoniumbicarbonate (TEAB) 50 mM mixed with Trypsin + LysC (2 μg) was added to the beads and incubated overnight (ON) at 37°C with 1000 rpm mixing for protein enzymatic digestion. Next, 1.3 mL of 100% acetonitrile (ACN) was added to samples and incubated for 20 minutes at 22°C with 1000 rpm mixing. Tubes were placed in a magnetic rack, and beads were washed with 100% ACN. A hundred μL of TEAB 50 mM was added to the beads, resuspended, and incubated for 5 minutes at 22°C with 1000 rpm mixing. After incubation, tubes were placed in a magnetic rack until the beads had migrated to the tube wall, and the SN was transferred to a new tube with 20 μL of 5% formic acid (FA). Afterward, tubes with FA were placed in a SpeedVac until the samples were dry. Then, samples were resuspended in FA 0.1%, and peptides were quantified by Pierce^™^ Quantitative Fluorometric Peptide Assay (Thermo Fisher Scientific, USA). Five hundred ng of peptides of each sample were analyzed by LC.MS.

### LC-MS/MS analysis

Protein identification and quantitation were performed by nanoLC–MS/MS, using an Ultimate 3000 liquid chromatography system coupled to a Q-Exactive Hybrid Quadrupole-Orbitrap mass spectrometer (Thermo Fisher Scientific, Bremen, Germany). Samples were loaded onto a trapping cartridge (Acclaim PepMap C18 100Å, 5 mm x 300 μm i.d., 160454, Thermo Fisher Scientific) in a mobile phase of 2% ACN, 0.1% FA at 10 μL min^−1^. After 3 minutes of loading, the trap column was switched in-line to a 50 cm by 75μm inner diameter EASY-Spray column (ES803, PepMap RSLC, C18, 2 μm, Thermo Fisher Scientific) at 250 nL minute^−1^. Separation was generated by mixing A: 0.1% FA, and B: 80% ACN, with the following gradient: 5 minutes (2.5% B to 10% B), 120 minutes (10% B to 30% B), 35 minutes (30% B to 50% B), 3 minutes (50% B to 99% B) and 12 minutes (hold 99% B). Data acquisition was controlled by Xcalibur 4.0 and Tune 2.9 software (Thermo Scientific, Bremen, Germany).

The mass spectrometer was operated in data-dependent (dd) positive acquisition mode alternating between a full scan (m/z 380-1580) and subsequent HCD MS/MS of the 10 most intense peaks from full scan (normalized collision energy of 27%). ESI spray voltage was 1.9 kV. Global settings: use lock masses best (m/z 445.12003), lock mass injection Full MS, chrom. peak width (FWHM) 15s. Full scan settings: 70k resolution (m/z 200), AGC target 3e6, maximum injection time 120 ms. dd settings: minimum AGC target 8e3, intensity threshold 7.3e4, charge exclusion: unassigned, 1, 8, >8, peptide match preferred, exclude isotopes on, dynamic exclusion 45s. MS2 settings: microscans 1, resolution 35k (m/z 200), AGC target 2e5, maximum injection time 110 ms, isolation window 2.0 m/z, isolation offset 0.0 m/z, spectrum data type profile.

### Data and Bioinformatics Analysis

The acquired raw data were analyzed using the MaxQuant search engine 1.5.3.30 (29) for protein identification and label-free quantification (LFQ) (iBAQ intensity analysis). The data were searched against the UniProt human database (UP000005640, downloaded October 2020). The search parameters were set with the following conditions: two missed cleavage sites for trypsin/P, carbamidomethylation of cysteine as fixed modification, oxidation of methionine and acetylation of N-terminal residue as variable modifications, and precursor ion mass tolerance of 20 ppm. The false discovery rate (FDR) of peptides and proteins was set to 1%.

Software Perseus 1.6.14.0 (30) was used for data processing and statistical analysis. Reverse, contaminant, and only identified by site proteins were excluded, and iBAQ values were log_2_ transformed. Missing values were imputed by replacing missing values with a constant (=1). All datasets were subjected to a student’s t-test and p-values less than 0.05 with fold-change higher than 2 were considered statistically significant.

Gene Ontology (GO) pathway analysis was performed using DAVID Bioinformatics (31). For these analyses, a p-value cutoff of 0.05 was applied. All significant pathways were extracted from the GO category, but only the top 5 most significant terms are represented in the graphs. Network analysis was performed with Search Tool for the Retrieval of Interacting Genes/Proteins (STRING) (32). To avoid making the network too complicated, high confidence (=0.7) was applied for both up- and down-regulated proteins, and interaction sources considered were textmining, experiments, gene fusion, databases, co-occurrence, and co-expression. The acquired raw data were also analyzed using Virtual Expert Mass Spectrometrist software (33) for the identification of Post-Translational Modifications (PTM). Peptide mass accuracy tolerance was set to 5 ppm and fragment mass accuracy to 0.01 Da. Carbamidomethylation of cysteine as fixed modification, oxidation of methionine, and acetylation of N-terminal residue as variable modifications. The following modifications were considered for lysine: diglycine tag for ubiquitination, acetylation, mono-, di- and tri methylation were included as variable modifications. In addition, serine, threonine, and tyrosine phosphorylation were included as variable modifications. FDR threshold was set to 1%.

### Histone acid extraction

Histones were extracted by an acid-extraction method (adapted from (34)). Briefly, cells were washed twice with ice-cold PBS and then resuspended in Triton Extraction Buffer (TEB: PBS containing 0.5% Triton X-100 (v/v), 2mM phenylmethylsulfonyl fluoride, 0.02% (w/v) NaN_3_). TEB volume added was adjusted to cell density (1 mL of TEB per 10^7^ cells). Cells were lysed on ice for 10 minutes with gentle stirring and then centrifuged at 650g for 10 minutes at 4°C to spin down the nuclei. The supernatant was discarded, and nuclei were washed with half the volume of the initial TEB through a new centrifugation. The pellet was then resuspended in 0.2N HCl, and histone acid was extracted ON at 4°C. HCl volume added was adjusted to cell density (1 mL of HCl per 4×10^7^ cells). Samples were further centrifuged at 650g for 10 minutes at 4°C to pellet debris, the supernatant was collected, and HCl was neutralized with 2M NaOH (1/10 of the volume of the supernatant). Protein concentration was determined using the DCProtein assay kit from BioRad (California, USA). The extracted proteins were stored at −20 °C until further analysis.

### Western blotting

Fifteen μg of total protein or 1–3 μg of nuclear proteins were separated via 4–20% sodium dodecyl sulfate‒polyacrylamide gel electrophoresis under denaturing conditions and subsequently transferred to Hybond ECL membranes (Amersham Biosciences, GE Healthcare, UK). The membranes were blocked for 1 hour at RT and incubated ON with the respective primary antibodies (Table S1) at 4°C with agitation. After incubation with specific anti-rabbit or anti-mouse horseradish peroxidase-conjugated secondary antibodies for 1 h at RT, the signal was visualized with Clarity Western ECL Blotting Substrate (Bio-Rad, California, USA) and detected with a Bio-Rad ChemiDoc XRS System. For histones, the membranes were also incubated with specific anti-rabbit secondary antibodies for 1 hour at RT, and the signal was detected using an Odyssey CLx Infrared Imaging System. Band intensity was quantified using ImageJ.

### Transmission electron microscopy (TEM)

For the ultrastructure analysis, cells were fixed in a solution of 2.5% glutaraldehyde (Electron Microscopy sciences) with 2% formaldehyde (Electron Microscopy sciences) in 0.1 M sodium cacodylate buffer (pH 7.4) for 1 hour, at RT, and post-fixed in 1% osmium tetroxide (Electron Microscopy Sciences) diluted in 0.1 M sodium cacodylate buffer. After centrifugation, the pellet was resuspended in HistogelTM (Thermo) and then stained with aqueous 1% uranyl acetated solution ON, dehydrated, and embedded in Embed-812 resin (Electron Microscopy sciences). Ultra-thin sections (50 nm thickness) were cut on a RMC Ultramicrotome (PowerTome, USA) using Diatome diamond knives, mounted on mesh copper grids (Electron Microscopy Sciences), and stained with uranyl acetate substitute (Electron Microscopy Sciences) and lead citrate (Electron Microscopy Sciences) for 5 minutes each. Samples were visualized on a JEOL JEM 1400 transmission electron microscope (JEOL, Tokyo, Japan), and the images were digitally recorded using a CCD digital camera Orius 1100W (Tokyo, Japan).

### TEM image analysis

Following standard TEM preparation and staining protocol, heterochromatic regions are heavily stained while euchromatic regions are lightly stained. The image contrast at the bright field for a thin resin section follows Beer’s Law, which can be converted to DNA concentration with calibration:

(1) $$I\left(x,y\right)=I_{0}e^{-\sigma \rho \left(x,y\right)t}$$

Here $I\left(x,\;y\right)$ is the intensity of the resultant image, $I_{0}$ is the intensity of the incident beam, σ is the absorption coefficient of the sample, $\rho \left(x,y\right)$ is the density distribution, and t is the thickness of the section consisting of the sample. We assumed that for a given resolution, the absorption coefficient is constant. Further, the incident beam and section thickness were controlled to be the same across all images. Therefore, after taking a negative logarithm of the image, mean intensity was measured, representing the heterochromatin amount.

### RNA extraction and Sequencing

For the analysis of RNA expression at 72 hours after KRAS silencing, total RNA extraction was performed using TRIzol^™^ Reagent, according to manufacturer’s instructions (Thermo Fisher Scientific, Waltham, MA, USA). Briefly, spheroids were removed from the molds, subjected to centrifugation at 1200 rpm for 5 minutes, and the supernatant was discarded. TRIzol^™^ Reagent was added to the spheroids, and the samples were homogenized, allowing them to stand for 5 minutes at room temperature. Subsequently, 1-Bromo-3-chloropropane (BCP) was introduced, and the samples were shaken before standing at RT for an additional 5 minutes. The samples were then centrifuged at 13200 rpm for 15 minutes at 4°C. Following centrifugation, the aqueous phase was carefully removed and transferred to a fresh tube. Isopropanol was incorporated to precipitate RNA, with the tubes being inverted several times for thorough mixing, followed by a 10-minute incubation at RT. After centrifugation at 13200 rpm for 10 minutes at 4°C, the supernatant was meticulously discarded, and the RNA pellet was washed with 75% EtOH. After another centrifugation at 10,000 rpm for 5 minutes at 4°C, the supernatant was removed, and the pellet was air-dried for 5 minutes. The pellet was then solubilized by adding DEPC-treated water and incubating at 60°C for 10 minutes. Purification of RNA was accomplished using the RNeasy MinElute Cleanup Kit (Qiagen) in accordance with the manufacturer’s protocol. Library preparation and sequencing was performed by Northwestern University NUSeq Core Facility. Library construction was performed following TruSeq mRNA-Seq library preparation. Illumina HiSeq 4000 Sequencer was used to sequence the libraries with the production of single-end, 50–base pair reads.

For analysis of RNA expression at different timepoints (12, 24, 48 and 72 hours after siRNA transfection), total RNA was extracted using a mirVana^™^ miRNA Isolation Kit (Thermo Fisher Scientific, USA) according to the manufacturer’s instructions. Library preparation and sequencing were performed by Novogene Europe. Briefly, for library construction, messenger RNA was purified from total RNA using poly-T oligo-attached magnetic beads. Libraries were sequenced on an Illumina NovaSeq 6000 platform using a paired-end 150-base pair strategy.

### RNA sequencing data analysis

The quality of the reads was evaluated using FASTQC with fastq files. Adapters were trimmed and reads of poor quality or aligning to rRNA sequences were filtered out using Trimmomatic. The cleaned reads were aligned to the human genome (hg38) using STAR. Counts and transcripts per million for each condition were estimated from mapped reads using RSEM. All the data were processed using RStudio version 2023.06.0.

For RNA sequencing from 72h time-point, differential expression was determined using DESeq2. The cutoff for determining significantly differentially expressed genes was an FDR-adjusted p-value less than 0.05 and log2 fold-change <=−1 or =>1.

Differentially expressed genes for RNA-Sequencing at several time-points were determined using the NOISeq R package. NOISeq is a novel nonparametric approach for identifying differentially expressed genes from RNA-Seq count data. This method was designed to compute the differential expression of RNA-Seq data even when there were no replicates available for any of the experimental conditions. GO biological process (BP) pathway analysis was performed using DAVID Bioinformatics. All BPs were extracted from the GO category, but only the 5 most significant terms of up- or downregulated genes are represented in the graphs.

### Quantification of G0 arrest

G0 arrest was quantified using the abovementioned RNA-Seq data by the G0 arrest score quantification methodology described in Wiecek, A.J. *et al.* (35). The gene lists representing quiescence programs and the code used for G0 arrest score quantification are available at https://github.com/secrierlab/CancerG0Arrest.

### Sample preparation for PWS Microscopy

To prepare spheroids for PWS microscopy, the media was removed from molds, and subsequently, spheroids underwent fixation with 2% PFA and 0.05% GA for 1 hour at RT. After fixation, the spheroids were washed thrice with PBS. A layer of 1% agarose was applied over the molds, and they were then embedded in paraffin. The paraffin-embedded spheroids were sectioned into 4 μm sections by the Pathology Core Facility at Northwestern University, and these sections were placed onto slides.

The deparaffinization protocol was carried out on the slides as follows: the slides were heated for 10 minutes at 50°C, washed overnight in xylene, rinsed with a 50/50 xylene/ethanol solution for 5 minutes, and then consecutively rinsed in ethanol (100%, 75%, 50%, and 25%) for 5 minutes each. Finally, the slides were washed with water and mounted on hollow-bottom dishes for imaging. Prior to imaging, the samples were stained with DAPI (1:1000 in PBS) for 10 minutes at RT.

### PWS Microscopy

Briefly, PWS measures the spectral interference signal resulting from internal light scattering originating from nuclear chromatin. This is related to variations in the refractive index distribution (Σ) (extracted by calculating the standard deviation of the spectral interference at each pixel), characterized by the chromatin packing scaling (D). D was calculated using maps of Σ, as previously described(36–39). Measurements were normalized by the reflectance of the glass medium interface (i.e., to an independent reference measurement acquired in a region lacking cells on the dish). This allows us to obtain the interference signal directly related to refractive index (RI) fluctuations within the cell. Although it is a diffraction-limited imaging modality, PWS can measure chromatin density variations because the RI is proportional to the local density of macromolecules (e.g., DNA, RNA, proteins). Therefore, the standard deviation of the RI (Σ) is proportional to nanoscale density variations and can be used to characterize packing scaling behavior of chromatin domains with length scale sensitivity around 20 – 200 nm, depending on sample thickness and height.

The PWS microscopy images were acquired on a commercial inverted microscope (Leica, Buffalo Grove, IL, DMIRB) with a Hamamatsu Image EM charge-coupled device camera (C9100–13) coupled to a liquid crystal tunable filter (CRi, Woburn, MA) to collect spectrally resolved images between 500 to 700 nm with 1 nm step size. Further, broadband illumination is provided by an Xcite-120 LED lamp (Excelitas, Waltham, MA). Samples were prepared as described before, and imaged with PBS. At least 40 cells were quantified from each condition in each experiment and three biological replicates were used for the analysis.

### Preparation of Hi-C Libraries

Hi-C was generated using the Arima-HiC Kit, according to the manufacturer’s protocols. Briefly, around 5 million cells were collected from spheroids and pellet by centrifugation at 1200rpm for 5 minutes. Cells were then crosslinked with 2% formaldehyde for 10 minutes at RT. Samples were incubated for 5 minutes at RT with Stop Solution 1 to quench the reaction, and then incubated on ice for 15 minutes. Samples were centrifuged at 1200rpm for 5 minutes and resuspended in PBS. After centrifugation at 1200rpm for 5 minutes, cell pellets were flash-frozen in liquid nitrogen and stored at −80°C. Nuclei were permeabilized with lysis buffer for 30 minutes at 4°C. Conditioning solution was added to the permeabilized nuclei and incubated at 62°C for 20 minutes. Reaction was quenched adding stop solution 2 and incubated for 15 minutes at 37°C. DNA was digested using consecutive enzymes mix and different incubation times and temperatures, as explained in manufacturers protocol. Library preparation was performed using the Arima-HiC Kit – KAPA^®^ Hyper Prep Kit, according to the manufacturer’s protocols. First, DNA was fragmented to a length of ~400bp using a LE220-plus Focused-ultrasonicator (Covaris). Then, DNA size selection was performed with AMPure XP Beads. After, DNA was quantified using the Qubit dsDNA High Sensitivity Assay Kit. Point ligation junctions were pulled down with enrichment beads.

Library preparation was performed using Illumina primers and protocol. Library amplification was accomplished following the manufacturers protocol. Paired-end sequencing was performed by Admera health using the Illumina HiSeq 2000 OR 2500 platform.

### Hi-C Data Processing and Analysis

Hi-C data was processed using Juicer tool (40). This tool uses the Burrows-Wheeler single end aligner (BWA) to map each read end separately to the hg38 reference genome, removes reads that map to the same fragment, removes duplicate or near-duplicate reads, and filters the remaining reads based on the mapping quality score. A/B compartments were estimated by eigenvector analysis at 500kb resolution of the Hi-C contact matrix. These two compartments have previously been found to be associated with open and closed chromatin; in the following, we will use open to refer to the A compartment and closed to refer to the B compartment. The sign of the eigenvector is arbitrary. Domains were annotated using Arrowhead at 10kb resolution, and loops were annotated using HiCCUPS. Juicebox was used to visualize Hi-C contact maps.

### Statistical analysis

GraphPad Prism version 9.0 (GraphPad Software, Inc., USA) was used for the statistical analysis of the data. Normality of samples was tested using Shapiro-Wilk test. Paired t tests and two-way ANOVA or nonparametric equivalent tests were used according to the most adequate test for each comparison. The test used for each comparison is indicated in the figure legends. Significance was defined as a p-value*≤0.05, p-value**≤0.01, p-value*** ≤0.001, and p-value**** ≤0.0001.

### Data availability statement

RNA-Seq data discussed in this publication have been deposited in NCBI’s Gene Expression Omnibus (GEO) and are accessible through GEO Series accession number GSE249954, GSE254832 and GSE254833. The mass spectrometry proteomics data have been deposited to the ProteomeXchange Consortium via the PRIDE partner repository with the dataset identifier PXD047770. The remaining data generated in this work is contained within the article or supplementary material.

## Results

### KRAS dependency is retained in spheroids from colorectal cancer cell lines

Three mutant KRAS CRC cell lines were used in this study (HCT116, SW480, and LS174T). Despite all harboring KRAS mutations, in 2D cell culture models, the three cell lines were previously shown to display distinct degrees of dependency on KRAS oncogenic signaling to survive (41). To validate KRAS dependency in 3D cell cultures, 24 hours after KRAS inhibition, cells were seeded in 3D conditions and cultured for another 48 hours. As such, all the analyses were performed 72 hours after KRAS inhibition (Figure S1A-B).

**Figure 1:**
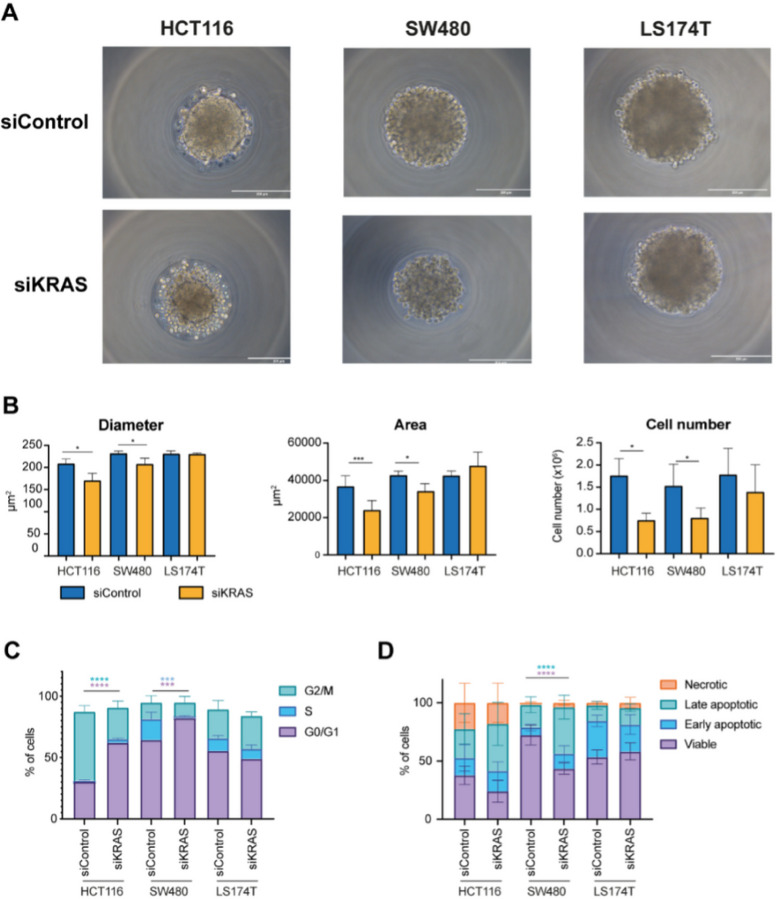
Colorectal cancer cells retain KRAS dependency in 3D cell cultures. HCT116, SW480, and LS174T cells, both siControl and siKRAS, were grown as spheroids for 48 hours. **A** Representative microscopy images of spheroids of siControl and siKRAS cells (scale bar: 200 μm). **B** Quantification of spheroids diameter, area and cell number; paired t-test was used for statistical analysis (*P≤0.05; ***P ≤0.001). **C** Cell cycle analysis: bar graphs illustrate the percentage of cells in the different cell cycle phases (G0/G1, S, G2/M); Two-way ANOVA with multiple comparisons was used to compare different populations in siControl with siKRAS conditions (*P≤0.05; **P ≤0.01; ***P ≤0.001). **D** Apoptosis analysis: bar graphs represent the percentage of cells that are in viable, early or late apoptosis, and necrotic statesTwo-way ANOVA with multiple comparisons was used to compare different populations in siControl with siKRAS conditions (*P≤0.05; **P ≤0.01; ***P ≤0.001; ****P ≤00001). All the bar graphs represent the mean ± SD of at least three independent experiments.

The three cell lines formed spheroids in less than 24 hours after seeding, either with a compact (HCT116) or loose (SW480 and LS174T) morphology (Figure 1A). The spheroid diameter at 48 hours after 3D seeding ranged from ~200–250 μm, depending on the cell line (Figure 1B). Although all the cell lines exhibited a high degree of KRAS silencing (Figure S1A), only HCT116 and SW480 cell lines exhibited KRAS-signaling dependency. In both cell lines, spheroids formed by siKRAS cells had a smaller area and diameter compared with spheroids formed by siControl cells, along with a reduction in the number of cells (Figure 1B). Additionally, HCT116 siKRAS spheroids tended to exhibit decreased circularity and a significant decrease in solidity (Figure S2). No significant alterations were observed between the siControl and siKRAS LS174T spheroids (Figure 1A–B), further corroborating the independence of KRAS signaling of this cell line.

Next, we evaluated the impact of KRAS silencing on cell cycle progression and apoptosis by flow cytometry. The gating strategy used for cell cycle and apoptosis analysis can be found as supplementary data (Figure S3). Compared with siControl cells, siKRAS HCT116 cells exhibited a significant increase in the G0/G1 phase and a decrease in the G2/M phase. siKRAS SW480 cells exhibited a significant increase in the G0/G1 population and a decrease in the S phase population (Figure 1C) compared with their respective siControl. Regarding apoptosis, statistically significant differences were only detected in siKRAS SW480 cells which showed a decrease in viable cells and an increase in late apoptotic cells compared with siControl cells (Figure 1D). No differences were observed for KRAS-signaling independent LS174T cells, either in terms of cell cycle or apoptosis (Figure 1C–D).

Furthermore, we calculated the G0 arrest score for both siControl and siKRAS cells using an algorithm that defines a G0 arrest score derived from transcriptomic data (35). When evaluating the G0 arrest score based on the established generic signature, we observed that independent of the cell line analyzed, siKRAS cells presented a positive score, thus indicating the induction of a G0 arrest signature (Figure S4). Notably, the G0 arrest score of siKRAS HCT116 and SW480 cells was higher than the LS174T score.

In summary, our results confirm that SW480 CRC cells are sensitive to KRAS silencing, and LS174T cells are resistant. HCT116 cells, although originally classified as KRAS-independent based on survival analysis in 2D cell cultures, are herein classified as sensitive to KRAS silencing based on the proliferation and quiescence results.

### CRC cells sensitive to KRAS silencing exhibit a proteomic profile indicative of chromatin and transcriptional reprogramming

To dissect the main cellular programs altered upon KRAS silencing, we performed a proteomic analysis in siControl and siKRAS CRC spheroids. Principal component analysis revealed a complete discrimination between siControl and siKRAS cells in HCT116 and SW480 (Figure S5), while in LS174T, that discrimination was not observed (Figure S5). Despite these observations, no significant changes were observed in the number of identified proteins between siControl and siKRAS cells in all the cell lines (Figure S5 and Table S2).

**Figure 2:**
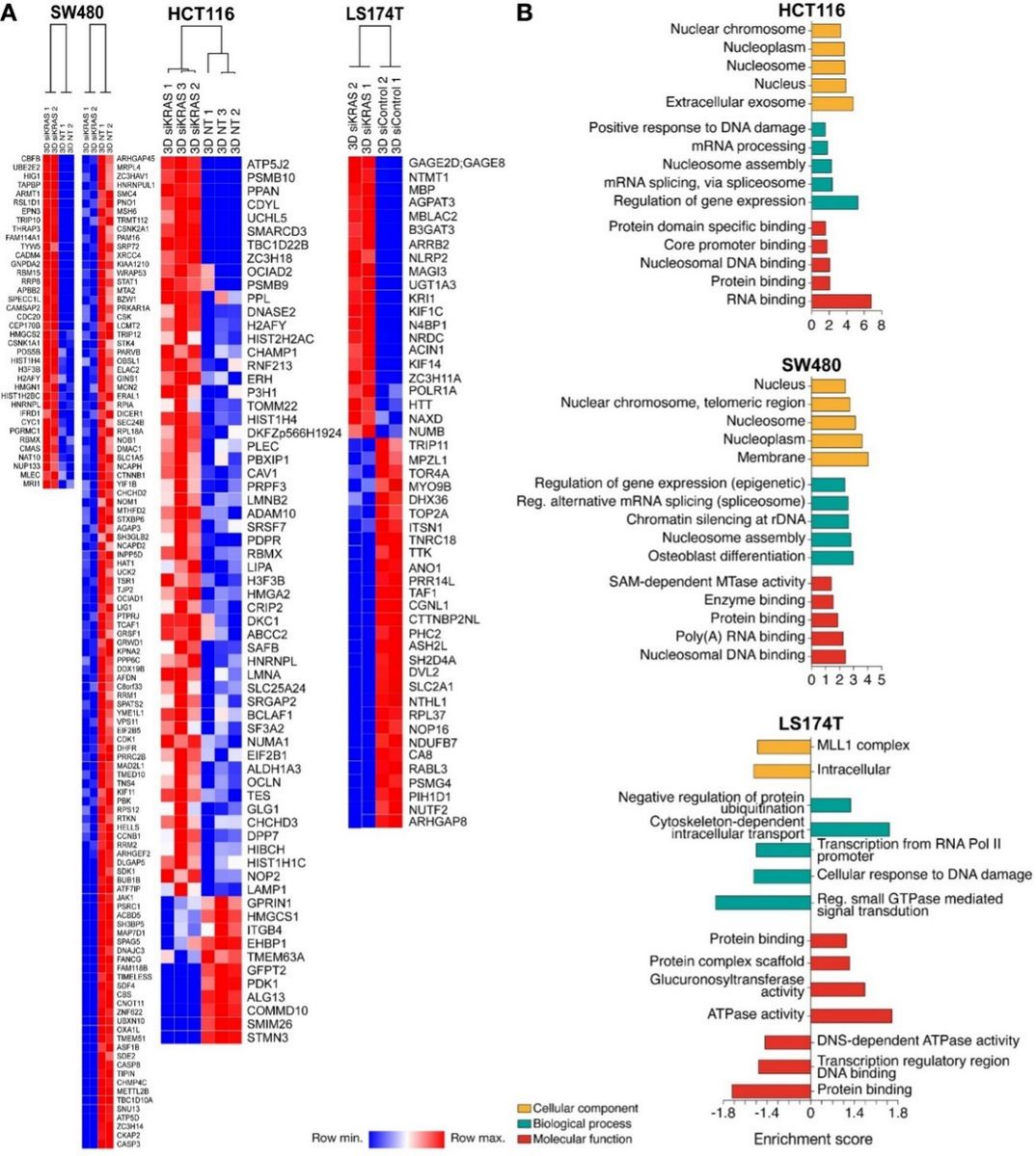
Spheroids from KRAS-dependent silenced cells exhibit a proteomic profile indicative of chromatin reprogramming. **A** Heatmap of up- and down-regulated proteins. Proteomic analysis was performed in three biology replicates of HCT116 and two biologic replicates of SW480 and LS174T CRC cells. **B** Gene ontology (GO) enrichment analysis of differently expressed proteins between siKRAS and siControl cells; the up-regulated and down-regulated proteins were classified according to their cellular component (yellow bars), biological processes (green bars) and molecular function (red bars); The enrichment score of GO terms was calculated by −log p-value; the graphs show the top 5 GO terms sorted by p-value.

We observed that a total of 55 proteins were upregulated and 11 proteins were downregulated in siKRAS HCT116 cells; 38 proteins were upregulated and 113 were downregulated in siKRAS SW480 cells; 21 proteins were upregulated and 29 were downregulated in siKRAS LS174T cells compared with the respective siControl cells (Figure 2A and Table S2). The difference in the expression of randomly selected proteins between the siControl and siKRAS cells was validated by western blotting (Figure S6).

GO analyses were performed to classify the proteins differentially expressed between siControl and siKRAS cells by cellular component, biological processes, and molecular function. In the two KRAS-silencing sensitive cell lines, HCT116 and SW480, upregulated proteins were mainly localized at nuclear compartments (nucleus, nucleosome, and nucleoplasm), extracellular exosome, and in the membrane (Figure 2B). Upregulated proteins in LS174T cells were not associated with any cellular component. For the downregulated proteins, the cellular component category was only possible to be determined in SW480 and LS174T cells. In SW480, proteins were mainly localized in cytosol and cytoplasm (Table S3), while in LS174T cells, proteins were mainly localized intracellularly and in the MLL1 complex (Figure 2B). The biological processes category revealed that upregulated proteins were associated with several processes in HCT116 and SW480 cells, including regulation of gene expression, mRNA splicing, nucleosome assembly, and mRNA processing, among others, while in LS174T cells were only associated with intracellular transport and regulation of protein ubiquitination. Downregulated proteins in HCT116 were mainly associated with neuron projection development, cell division and cell-cell adhesion in SW480, and signal transduction, response to DNA damage, and transcription in LS174T (Table S3 and Figure 2B). Molecular function GO terms for upregulated proteins were mainly associated with binding activities (RNA, protein, nucleosome, and core promoter binding) in HCT116 and SW480 cells, and associated with ATPase activity in LS174T cells. For the downregulated proteins, it was only possible to obtain information for SW480 and LS174T, and in both, they were associated with binding activities (Figure 2B and Table S3).

STRING was used to determine possible protein-protein interactions among the proteins differentially expressed between siControl and siKRAS cells (Figure S7). Proteins that were upregulated in HCT116 siKRAS cells formed two major clusters: one of the clusters was composed of proteins associated with the nucleosome, including HMGA2, HIST1H1C, HIST1H4F, HIST2H2AC, H3F3B and H2AFY; the other cluster was composed of mRNA splicing-related proteins, such as SF3A2, PRPF3, RBMX, SRSF7, SAFB and HNRNPL (Figure S7A). In SW480 siKRAS cells, upregulated proteins formed a cluster associated with nucleosomes that included proteins such as H3F3B, HIST1H4F, HIST1H2BC, and H2AFY and a cluster associated with chromosome condensation formed by downregulated proteins such as SMC4, NCAPD2, CCNB1, CDK1, PBK, DLGAP5, CKAP2, SPAG5 and KIF11 (Figure S7B). Proteins differentially expressed in LS174T siKRAS cells did not exhibit significant interactions and did not show any specific clustering (Figure S7C).

In summary, KRAS silencing significantly impacted the cellular proteome, exhibiting distinct effects on KRAS-silencing sensitive and resistant cell lines. KRAS silencing in sensitive cells enhanced proteome remodeling within the nuclear compartment, influencing the expression of proteins associated with chromatin and the regulation of gene expression.

### KRAS silencing impacts the physical organization of chromatin

Given that a significant number of the upregulated proteins in KRAS-silencing sensitive cell lines were localized within the nucleus (such as the nucleoplasm, nucleolus, and chromosomes) and that the affected biological processes were associated primarily with chromatin reorganization, nucleosome assembly, and gene expression, we further aimed to characterize the status of chromatin organization in siControl and siKRAS cells.

Histone post-translational modifications (PTMs) play a fundamental role as epigenetic regulators of chromatin accessibility and gene expression. A recent study showed that the induction of mutant KRAS increased the global expression levels of histone marks associated with active transcription (H3K27ac and H3K4me3) and with silent chromatin (H3K27me3 and H3K9me3)(42). Therefore, we explored our proteomic data for alterations in histone PTMs to assess whether this could be a mechanism involved in chromatin remodeling. Acetylation of lysine 18 and 23 in histone 3 (H3k18ac, H3k23ac) and acetylation of lysine 8, 12, and 16 in histone 4 (H4k8ac, H4k12ac, H4k16ac), marks associated with active transcription, were found in KRAS-silenced sensitive cell lines (Figure S8). Acid extraction of nuclear histones was performed to validate these PTMs, as well as other PTMs that, although not identified in our proteomics experiments, are commonly associated with regulatory elements. These included histone marks of enhancers and super-enhancers (H3k27ac), active promotors (H3k9ac, H3k4me3), transcriptionally active gene bodies (H3k36me3), and silent chromatin (H3k27me3). Through western blotting, we were not able to validate H3K18ac found in the proteomics (Figure S9A-B). For the other PTMs, only HCT116 siKRAS cells showed a significant decrease in H3k9ac. Besides that, no other changes in the nuclear levels of histone 3 PTMs were found in siKRAS HCT116 and siKRAS SW480 (Figure S9A-B). The western blotting results for the LS174T cell line were inconclusive, as most of the histone marks had very weak signals despite the increased amounts of protein used (Figure S9C).

**Figure 3:**
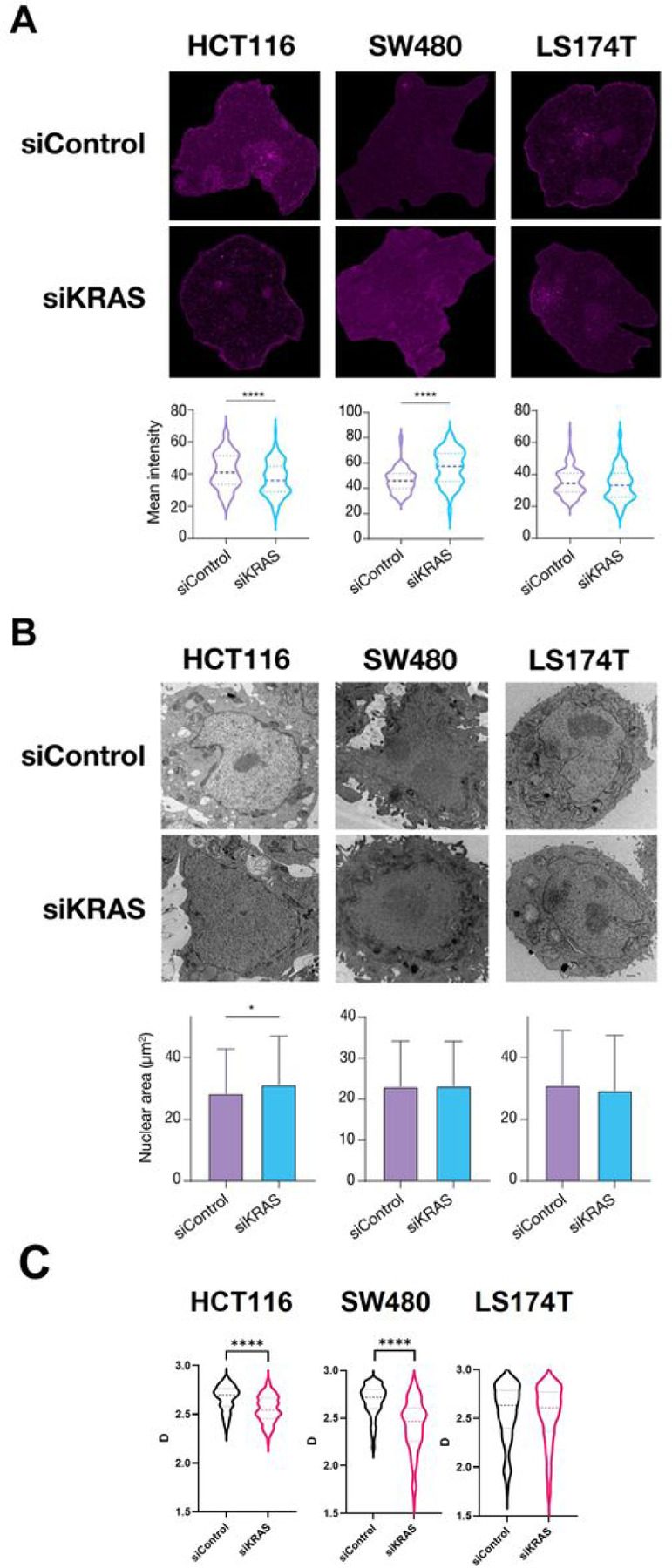
KRAS-dependent CRC cells show alterations at the level of chromatin compaction upon KRAS silencing. **A** Representative electron microscopy images showing areas of euchromatin (dark areas) and heterochromatin regions (bright purple), and respective Mean Intensity quantification of heterochromatin for each cell line. **B** Nucleus area quantified using Fiji. For quantification, three different experiments were performed and around 60 nucleus per condition were analyzed for each experiment. **C** Violin plots showing chromatin packing scaling (D) in siControl and siKRAS conditions of each cell line. The violin plots extend from the minimum to the maximum value. The line in the middle of each plot is the median value of the distribution, and the lines above and below are the third and first quartiles, respectively. Data was obtained from three biological replicates for each condition. Mann-Whitney test was used to perform all analysis; *P≤0.05, **P ≤0.01, ***P ≤0.001, ****P ≤0.0001.

Furthermore, the global proteomic changes identified in our study suggested that KRAS silencing may affect the higher-order packing state of chromatin. Higher-order packing of chromatin is intimately linked to overall transcription regulation, though it encompasses hierarchical compartmentalization of the genome into subdomains at different genomic scales. Therefore, to test if KRAS silencing alters chromatin organization, we started by using transmission electron microscopy (TEM) to characterize at the nuclear scale the global levels of euchromatin and heterochromatin, traditionally associated with active and repressed transcription, respectively. Seventy-two hours after KRAS silencing, the two siKRAS-sensitive cell lines exhibited alterations in chromatin packing, whereas no alterations were found in the siKRAS-resistant cell line (Figure 3A). Although the levels of heterochromatin were similar in siControl cells of the two sensitive cell lines, upon KRAS silencing, the chromatin packing state underwent opposing trajectories. In HCT116 siKRAS cells, the level of heterochromatin decreased concomitantly with an increase in the nuclear area; in turn, SW480 siKRAS cells displayed an increase in heterochromatin without changes in the nuclear area (Figure 3A–B).

Inside the nucleus, chromatin organizes into several thousand packing domains of variable sizes, densities, and fractal-like internal conformation, which influence the accessibility of chromatin to transcription regulators (38,43). The capacity of cell to form new packing domains and the chromatin conformation within and outside of packing domains within the nucleus was recently demonstrated to have a strong influence on global gene expression patterns and the adaptation and survival of cancer cells to chemotherapy. This phenomenon can be quantified by the average nuclear chromatin domain packing scaling. Specifically, higher chromatin packing scaling corresponds to packing domain upregulation and/or increased fractality of their internal conformation and heterogeneity thus engendering higher transcriptional malleability, heterogeneity, and plasticity (39,43). Conversely, packing domain downregulation is indicative of a shift of chromatin structure towards less plastic and dormant states. In order to evaluate whether cells respond to KRAS silencing by changing the 3D physical organization of their genome within the nucleus, we performed partial wave spectroscopic microscopy (PWS) to characterize the chromatin packing scaling on fixed spheroids. Our results showed a decrease in the packing scaling of chromatin upon KRAS silencing in the two siKRAS-sensitive cell lines, HCT116 and SW480 cell lines, whereas no changes were found in siKRAS-resistant LS174T cells when compared with the respective siCTRL cells (Figure 3C).

**Figure 4:**
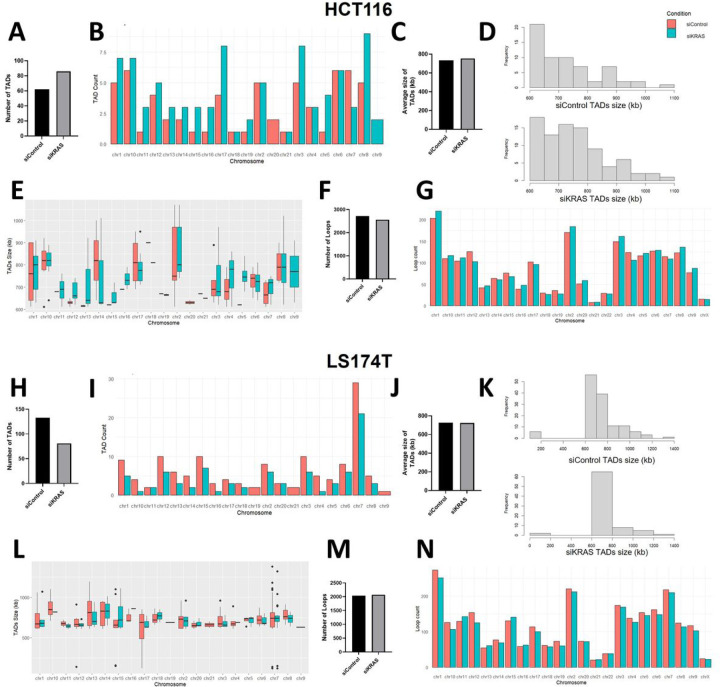
Hi-C shows alterations of genome-wide chromatin interactions upon KRAS inhibition. **A** Graph represents the number of TADs identified in HCT116 cells. **B** Graphs represent the number of TADs identified per chromosome in siControl and siKRAS HCT116 cells. **C** Graph represents the average size of TADs identified in HCT116 cells. **D** Graphs represent the frequency distribution of TADs size in siControl and siKRAS HCT116 cells. **E** Graphs represent the size of TADs identified in each chromosome in HCT116 cells. **F** Graph represents the number of loops identified in HCT116 cells. **G** Graphs represent the number of loops identified per chromosome in siControl and siKRAS HCT116 cells. **H** Graph represents the number of TADs identified in LS174T cells. **I** Graphs represent the number of TADs identified per chromosome in siControl and siKRAS LS174T cells. **J** Graph represents the average size of TADs identified in LS174T cells. **K** Graphs represent frequency distribution of TADs size in siControl and siKRAS LS174T cells. **L** Graphs represent the size of TADs identified in each chromosome in LS1774T cells. **M** Graph represents the number of loops identified in LS174T cells. **N** Graphs represent the number of loops identified per chromosome in siControl and siKRAS LS174T cells.

**Figure 5:**
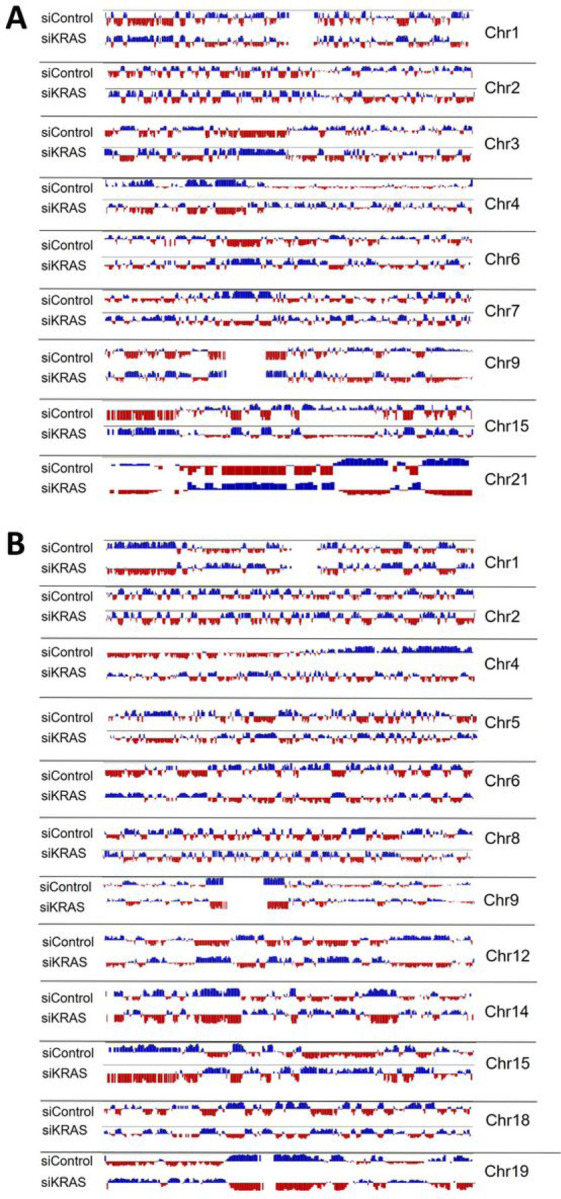
Hi-C shows alterations of A/B compartment upon KRAS inhibition. **A** Figure displays data of A/B compartment distribution in several chromosomes in siControl and siKRAS conditions for HCT116 cells. **B**. Figure displays data of A/B compartment distribution in several chromosomes in siControl and siKRAS conditions for LS174T cells. Data was obtained through eigenvector.

At a supranucleosomal scale, chromatin organizes into topologically associated domains (TADs) that bring together enhancers and promoters, thus demarcating an environment for preferred interactions (44). Loops are formed within TADs to mediate specific enhancer-promoter interactions (45). To map the alterations in genome-wide DNA interactions induced by KRAS silencing, we used high‐throughput chromosome conformation capture sequencing (Hi-C) in one siKRAS-sensitive (HCT116) and one resistance (LS174T) cell line. For the HCT116 cell line, more than 60 million contacts were generated for siControl (64,458,010) and siKRAS cells (70,658,657). In LS174T similar numbers were obtained, with 70,904,760 million contacts generated for siControl and 60,276,286 contacts for siKRAS cells. Using arrowhead analysis, 62 TADs were identified on siControl HCT116 cells, while 86 TADs were found on siKRAS HCT116 cells (Figure 4A–B). The average size of TADs did not change from siControl (~735kb) to siKRAS (~754kb) HCT116 cells (Figure 4C). Nonetheless, changes in size frequency were observed. Specifically, siKRAS cells exhibited a higher prevalence of larger TADs compared to siControl cells (Figure 4C–E). In LS174T cells, the opposite was observed. Regarding the number of TADs, siKRAS cells showed a decrease in the number of TADs (Figure 4H–I) - 133 TADs were found on siControl LS174T cells, while 81 TADs were found on siKRAS LS174T cells (Figure 4H–I). The average size of TADs in LS174T cells did not change between siControl (~728kb) and siKRAS (~723kb) conditions (Figure 4J), and no major differences regarding size frequency were observed (Figure 4J–L). In addition to TADs, we also evaluated the number of chromatin loops. More than 2000 loops were identified in both cell lines in siControl or siKRAS conditions. Nonetheless, there were no major alterations in the total number of loops found between siControl or siKRAS (Figure 4F and Figure 4M), nor in the number of loops found per chromosome (Figure 4G and Figure 4N) in the two cell lines. Moreover, the Hi-C long-range contact frequency matrix also provides a distribution of the chromatin into A and B compartments. For instance, within chromosomes, chromatin segregates into large-scale A and B compartments corresponding to transcriptionally active and repressive regions, respectively (46). In the two studied cell lines, no alterations were found in the global levels of chromatin within each compartment between siControl and siKRAS conditions (Figure S10). However, systematic analysis of A/B compartment tracks per chromosome revealed regions in several chromosomes where the compartment identity shifted (Figure 5) in the siKRAS condition compared to the siControl in both cell lines.

Altogether, these observations provide evidence for the occurrence of chromatin structural changes as part of the cell response to KRAS silencing. The highest impact on chromatin was observed in siKRAS-sensitive cells, which, besides shifting A/B compartments and potentially gaining TADs, demonstrated alterations at the eu/heterochromatin levels and at the 3D spatial conformation of chromatin within the nucleus.

### KRAS silencing enhances transcriptional variability in sensitive cell lines

Although the relationship between TADs and gene expression is still controversial (47,48), we tested whether changes in TADs would impact the transcriptional profile of siKRAS cells. To do so, we performed RNA-sequencing (RNA-seq) in siControl and siKRAS HCT116 and LS174T cells at the same timepoint that Hi-C was performed. Differentially expressed genes between siControl and siKRAS were identified in both HCT116 and LS174T cells, revealing a higher number of DEGs in HCT116 than in LS174T cells. In particular, in HCT116 cells, 761 genes were found to be differentially expressed between siControl and siKRAS cells, with 267 genes up-regulated and 494 down-regulated in siKRAS cells (Table S4). In LS174T, a total of 457 genes were differentially expressed between siControl and siKRAS cells, 374 were up-regulated, and 83 were down-regulated in siKRAS cells (Table S4). As siKRAS HCT116 had an increase in the number of TADs, we checked whether the new TADs formed were contributing to the repertoire of differentially expressed genes. No association was observed as the majority of DEGs in siKRAS HCT116 cells were not located in the regions of the new TADs. Only two DEGs, TPBG (up-regulated) and ADRA1B (down-regulated), were located within new TADs. In LS174T cells, the same lack of association between DEGs and TAD loss was found.

**Figure 6:**
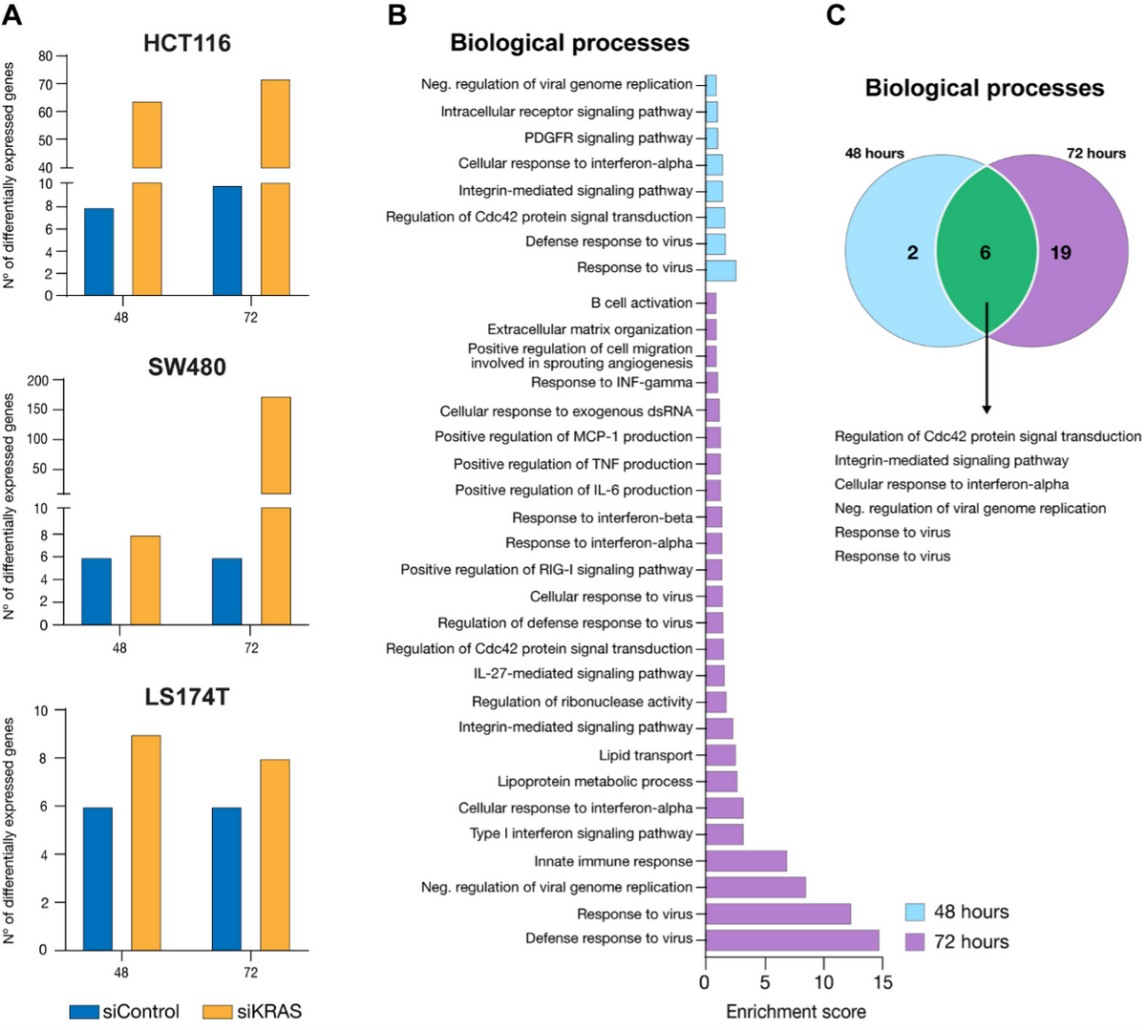
KRAS-dependent CRC cells exhibit higher transcriptional variability upon KRAS silencing. **A** Number of differentially expressed genes in siControl and siKRAS at the different time-points, compared with their respective 24-hour timepoint. **B** GO terms (biological processes) of differentially expressed genes between 48- and 72-hour and the 24-hour timepoint in siKRAS from HCT116 cells. The enrichment score was calculated by −log p-value. **C** Venn diagram illustrating the number of unique and shared biological processes altered at 48- and 72-hour timepoints in siKRAS HCT116 cells. Data is representative of one experiment. Differentially expressed genes were obtained through NOISeq R package, a tool that allows differential expression analysis from RNA-seq data with no replicates.

Recognizing the significance of transcriptional plasticity and heterogeneity in ensuring the survival and adaptation of cancer cells to stress (39,49) and the reported impact that chromatin packing scaling has on both (38,39), we then evaluated how variable the transcriptomic profile within siControl and siKRAS cells was. To do so, we performed RNA-seq at distinct timepoints (12−, 24−, 48−, and 72-hour) upon siRNA transfection in the two siKRAS-sensitive cell lines and in the one siKRAS-resistant. Although KRAS mRNA expression was highly reduced at 12 hours post-siRNA transfection (Figure S11A), protein downregulation was observed only at the 24-hour timepoint, and it was maintained throughout the following timepoints (Figure S11B-C). As such, we considered the 24-hour timepoint as the basal condition against which the subsequent timepoints (24- and 48-hour) were compared. This approach ensured that we did not compare conditions with highly disparate levels of KRAS protein. No significant differences were observed in the total counts between the different timepoints or between the siControl and siKRAS cells (Figure S12A). A multidimensional scaling plot revealed that the numbers of both siControl and siKRAS cells changed over time as they all spread throughout the plot (Figure S12B). However, it also revealed that siKRAS differ more between themselves over time than siControl cells. To determine whether siKRAS cells were transcriptionally more dynamic than siControl cells, thus resulting in higher transcriptional variability, we compared the number of differentially expressed genes (DEGs) from siKRAS cells at the 48- and 72-hour timepoints with that from the 24-hour timepoint (the first timepoint at which the maximum KRAS protein inhibition was reached). We also performed the same analysis in siControl cells. We observed that siKRAS-sensitive cell lines were transcriptionally more variable than siControl cells were, as evidenced by a greater number of DEGs over time (Figure 6A). Specifically, in HCT116 siKRAS cells, 64 and 72 genes were differentially expressed between the 24-hour timepoint and the 48- and 72-hour timepoints, respectively, whereas only 8 and 10 genes were differentially expressed at the same timepoints in siControl cells (Figure 6A). Most of the DEGs in the siKRAS cells were upregulated (53 and 64 were upregulated versus 11 and 8 were downregulated at the 48 and 72-hour timepoints, respectively) (Table S5). Additionally, only half of the DEGs were shared between the 48- and 72-hour timepoints (Figure S13A and Table S6), thus suggesting that transcriptional changes were not merely incremental but rather dynamic, with half of the genes being differentially regulated. Compared with siControl cells, SW480 siKRAS cells also demonstrated greater transcriptional variation, especially at 72 hours (Figure 6A). While only 8 genes were differentially expressed at the 48-hour timepoint compared to the 24-hour timepoint, 174 genes were differentially expressed at 72 hours. In the siKRAS-resistant cell line LS174T, the variations in the transcriptome of siKRAS cells was very similar to that of siControl cells, with very few genes exhibiting changes in expression between timepoints (Figure 6A). We then inquired whether the variability in gene expression across timepoints would lead to similar functional outcomes or alter the functional repertoire of the tolerant cells. To accomplish this, we conducted a Gene Ontology analysis to functionally characterize the genes differentially expressed in the two timepoints. This analysis was exclusively performed on HCT116 siKRAS cells due to their sufficiently high number of DEGs, which enabled the analysis at both timepoints. At 48 hours, eight biological processes were upregulated (Figure 6B and Table S5), including regulation of response to virus, intracellular and platelet-derived growth factor receptor signaling, response to interferon-alpha, integrin-mediated signaling, and regulation of CDC42 signal transduction (Figure 6B). At 72 hours, a total of 25 biological processes were upregulated: six were shared with the 48-hour timepoint, and 19 were acquired de novo (Figure 6B–C). Shared biological processes were mainly related to response to virus and regulation of CDC42 signaling. Within the acquired biological processes, we still observed a gain in processes related to response to virus, alongside processes associated with immune regulation, extracellular matrix remodeling, cell motility associated with angiogenesis, and lipid metabolism and transport. Therefore, we can conclude that the enhanced transcriptional variability observed in HCT116 siKRAS cells not only preserves certain functional outcomes but also introduces a diverse array of new functional possibilities.

In summary, the analysis of transcriptional activity revealed that siKRAS-sensitive cells exhibited a marked increase in transcriptional variability, resulting in heightened variability in gene expression and functional diversification across time. This emergent functional diversity may play a crucial role in the selection of resistant clones.

## Discussion

Cancer cells exhibit a remarkable capacity for adaptive resistance to treatments, particularly in the context of targeted inhibition of the oncogenic signal they rely upon (50). The clinical observations of enhanced tolerance to KRAS inhibition in CRC clearly typify this paradox. Although KRAS oncogenic activation is a well-established driver of CRC initiation and progression, its inhibition has not translated into clinical efficacy. Clinical data has revealed that the most common outcome in patients treated with KRAS inhibitors is stable disease (51), suggesting that while cancer cells are initially sensitive to KRAS inhibition, they rapidly develop tolerance. In our study, we took advantage of 3D in vitro models to shed light on the mechanisms underlying this rapid switch in sensitivity. Our findings demonstrate chromatin remodeling and enhanced transcriptional variability as potential epigenetic mechanisms enabling cancer cells to both tolerate and rapidly adapt to KRAS inhibition.

Herein, we used a strategy of KRAS inhibition through siRNA silencing of KRAS mRNA expression to investigate the mechanisms of acute adaptation of CRC cells to KRAS inhibition. This approach, although posing a limitation for long-term studies, captures the early response of cancer cells to KRAS loss, thus mimicking the initial phase of adaptation to KRAS-targeted inhibition. Moreover, it also allowed us to overcome the lack of inhibitors for the diversity of KRAS mutations that occur in CRC and the constraints in accessing the ones that have been recently developed. In addition, to better discern the mechanisms of tolerance to treatment, we used CRC cell lines with distinct sensitivities to KRAS silencing.

KRAS-silenced cells from the two KRAS-dependent cell lines were arrested in the G0/G1 phase of the cell cycle. This observation is consistent with the findings of preclinical and clinical studies, which showed that G0/G1 cell cycle arrest is a common feature of cancer cells after treatment with different KRAS inhibitors, including G12C, G12D, and pan-RAS inhibitors (52–54). Moreover, we also identified a transcriptomic signature of quiescence after KRAS silencing, which has previously been linked to therapeutic resistance (35). Despite diminishing tumor growth, cell cycle arrest and quiescence induced by KRAS silencing in KRAS-dependent CRC cells are likely to constitute a protective mechanism to survive KRAS inhibition. A timely transition to a quiescent-like, slow-cycling state, often associated with reversible G0/G1 cell cycle arrest, is a typical strategy applied by proliferative cancer cells to evade cell death when exposed to stress conditions. For example, metastatic cancer cells can remain dormant for long periods of time while adapting to new environments, and drug-sensitive proliferative cells switch to a drug-tolerant, slow-cycling state, a critical predecessor of therapy-resistant states (35,53,55).

The acquisition of a drug-tolerant phenotype can be achieved through reversible epigenetic reprogramming associated with transcriptional changes (56). Accordingly, quiescence and drug tolerance are transient states as proliferation and drug sensitivity can be recovered upon drug withdrawal (57,58). However, in the clinical setting, patients relapse while under treatment due to the emergence of therapy-resistant clones. In this case, quiescent drug-tolerant cells undergo genetic and/or epigenetic changes, endowing them with drug resistance and the capacity to resume growth (59). Understanding the molecular mechanisms underlying the drug-tolerant phenotype and the acquisition of resistance is fundamental, as it will delineate two distinct strategies that can be employed to improve the efficacy of treatments. On one hand, we may impair the transition to a quiescent state by forcing cells to continue proliferating under drug exposure, potentially leading to mitotic stress and cell death. On the other hand, we can prevent the emergence of resistant clones. In both cases, a better understanding of the biology of quiescent drug-tolerant cells is essential to identify actionable vulnerabilities. Our proteomics findings highlighted chromatin remodeling and regulation of transcription-associated processes as the main biological processes up-regulated in siKRAS-sensitive cells, alongside alterations in histone PTMs. Alterations of the global levels of histone PTMs towards more active or repressive marks could induce a global change in chromatin accessibility and transcription. However, histone PTM alterations were not validated by subsequent western blotting analysis. Although we cannot exclude the occurrence of spatial redistribution of specific histone PTMs as a consequence of any chromatin conformational alterations occurring in response to KRAS silencing, our findings lead us to rule out them as the main epigenetic drivers of chromatin remodeling. A more detailed characterization of the chromatin conformational state revealed alterations in the compaction and topological organization. Global nuclear analysis of chromatin compaction revealed that only siKRAS-sensitive cell lines changed their heterochromatin levels, whereas no changes were found in the siKRAS-resistant cell line. A more in-depth analysis through Hi-C, although revealing frequent shifts in A/B compartments in siKRAS-sensitive and resistant cell lines after KRAS silencing, showed a distinct trend regarding TADs formation: while the siKRAS-resistant cell line lost TADs, the sensitive cell line gained TADs which also tended to be larger. However, the comparison between the transcriptomic profile of siKRAS and siControl cells revealed that these changes in TADs were not yet contributing much to the differences in the transcriptional repertoire. Still, we cannot exclude that a gain in TADs may impact long-term adaptations of sensitive cells to KRAS silencing by expanding the array of possible enhancer-promoter interactions and, thus, their responsiveness to other stimuli. Our study also demonstrated that siKRAS-sensitive, but not siKRAS-resistant cells, adjusted the 3D physical organization of their genome within the nucleus after KRAS silencing by decreasing its packing scaling. This observation contrasts with the reported increase in chromatin packing scaling upon chemotherapy treatment (39,60). In this previous study, it was demonstrated that increased chromatin packing scaling influenced transcription malleability towards increasing the capacity of cancer cells to up-regulate initially overexpressed genes and suppress initially underexpressed genes. As such, through increasing chromatin packing scaling, cancer cells strengthen pre-existing pro-tumorigenic programs that allow them to survive the deleterious effect of chemotherapy. However, KRAS silencing, by itself, leads to a down-regulation of survival and proliferative signals that have been triggered by its oncogenic activation. Therefore, we hypothesize that, as cells are no longer actively proliferating, chromatin re-organizes towards decreasing the packing scaling to allow cells to find new transcriptional programs that allow them to survive and, later, resume growth independently of KRAS. This assumption is corroborated by longitudinal RNA-seq data demonstrating that siKRAS-sensitive cells had a higher transcriptional variability across timepoints than siControl cells. Therefore, our results indicate that siKRAS-sensitive cells enter a quiescent state, decrease their chromatin packing scaling, and become more transcriptionally variable, resulting in a diversification of functional processes, which may be a mechanism to rapidly adapt to the stress caused by KRAS inhibition. The observation that the siKRAS-resistant cell line, to whom KRAS inhibition does not pose any stress, maintains the original chromatin packing scaling and shows a lower transcriptional variability supports our assumption. Long-term studies complemented with single-cell analysis and lineage tracing will be pivotal for revealing how chromatin packing scaling and transcriptional variability co-evolve over time and impact the emergence and diversity of resistant clones. Additionally, pharmacological targeting of cancer cells’ ability to regulate chromatin packing scaling was previously demonstrated to be highly effective in enhancing the chemotherapy effects (60). Therefore, upcoming research will explore the potential benefits of combining KRAS inhibitors with agents targeting chromatin remodeling to enhance the overall efficacy of KRAS inhibition.

In conclusion, our findings suggest that the effects of KRAS inhibition extend beyond the immediate disruption of oncogenic signaling. Indeed, the stress response elicited by such targeted treatments appears to be mediated by a complex interplay of epigenetic mechanisms leveraged by 3D chromatin reorganization and transcriptional variability, which may underpin the remarkable ability of cancer cells to rapidly adapt, survive, and maintain malignancy even when deprived of oncogenic KRAS.

## Supplementary Material

Supplement 1
