# KRAS silencing alters chromatin physical organization and transcriptional activity in colorectal cancer cells

**DOI:** 10.21203/rs.3.rs-3752760/v3

**Published:** 2024-04-16

**Authors:** Flávia Martins, Ana Luísa Machado, Andreia Ribeiro, Susana Mendonça Oliveira, Joana Carvalho, Rune Matthiesen, Vadim Backman, Sérgia Velho

**Affiliations:** i3S - Institute for Research and Innovation in Health; i3S - Institute for Research and Innovation in Health; i3S - Institute for Research and Innovation in Health; i3S - Institute for Research and Innovation in Health; i3S - Institute for Research and Innovation in Health; Universidade Nova de Lisboa; Northwestern University; i3S - Institute for Research and Innovation in Health

**Keywords:** KRAS inhibition, drug tolerance, therapy resistance, chromatin packing scaling

## Abstract

Clinical data revealed that KRAS mutant tumors, while initially sensitive to treatment, rapidly bypass KRAS dependence to acquire a drug-tolerant phenotype. However, the mechanisms underlying the transition from a drug-sensitive to a drug-tolerant state still elude us. Here, we show that global chromatin reorganization is a recurrent and specific feature of KRAS-dependent cells that tolerated KRAS silencing. We show that KRAS-dependent cells undergo G0/G1 cell cycle arrest after KRAS silencing, presenting a transcriptomic signature of quiescence. Proteomic analysis showed upregulated chromatin-associated proteins and transcription-associated biological processes. Accordingly, these cells shifted euchromatin/heterochromatin states, gained topologically associating domains, and altered the nanoscale physical organization of chromatin, more precisely by downregulating chromatin packing domains, a feature associated with the induction of quiescence. In addition, they also accumulated transcriptional alterations over time leading to a diversification of biological processes, linking chromatin alterations to transcriptional performance. Overall, our observations pinpoint a novel molecular mechanism of tolerance to KRAS oncogenic loss driven not by specific gene alterations but by global reorganization of genomic information, in which cells transition chromatin domain structure towards a more quiescent state and gain transcriptional reprogramming capacity

## Introduction

KRAS stands out as the most mutated oncogene in cancer and occupies a pivotal position among the group of key cancer targets [1]. Mutations that render KRAS constitutively active occur in numerous cancer types at variable frequencies and prevail in lung (25%), colorectal (40%), and pancreatic (95%) cancers [2,3].

In colorectal cancer (CRC), mutant KRAS occurs at early stages of tumor development, and its oncogenic activity is needed for the progression from a premalignant to a malignant stage and for the development of metastasis [4–6]. Oncogenic KRAS signaling fuels cancer cells through strong mitogenic, survival, stemness, invasion, and prometastatic signals [5,7,8] and is also a major regulator of cancer cell metabolism [9]. KRAS mutations not only predict an unfavorable response to chemotherapy treatments such as folinic acid, fluorouracil, and oxaliplatin (FOLFOX) [10] but also serve as biomarkers indicating resistance to epidermal growth factor receptor (EGFR)-targeted therapies [11]. Mutant KRAS not only regulates crucial cancer activities but also extends its oncogenic signaling beyond cancer cells. It orchestrates a pro-tumorigenic crosstalk with fibroblasts, immune cells, and endothelial cells within the tumor microenvironment, thus expanding its repertoire of pivotal roles in cancer progression [12–14].

Based on the premise that the inhibition of such a multifaceted, central cancer driver would induce long-term anticancer responses, there have been numerous attempts to develop KRAS-targeted therapies [15]. This long-lasting endeavor is becoming fruitful, as the undruggable KRAS has recently become druggable. The first mutant-specific KRAS-targeted therapies were approved for the treatment of non-small cell lung cancer patients with G12C mutations [16], and many other inhibitors for other mutations or for the total protein are under development and/or in clinical trials [17–20].

Nevertheless, preclinical and clinical data have revealed that targeting KRAS mutant tumors is more challenging than initially expected, as cancer cells can rapidly bypass the dependence on this oncogene, thus recurring shortly after treatment initiation [21–23]. In CRC, stable disease occurs in most treated patients, suggesting that cancer cells, while initially sensitive to treatment, tolerate KRAS inhibition and maintain viability. Growth arrest lasts for approximately four to five months before disease progression occurs due to the emergence of resistance [22,24].

However, before resistance eventually develops, a subset of cells must persist in a drug-tolerant state during treatment [25,26]. Therefore, there is a compelling need to elucidate the mechanisms by which mutant KRAS cells withstand KRAS loss, as this understanding will offer valuable insights for the development of therapeutic strategies to improve the initial efficacy of KRAS inhibition. Specifically, targeting the mechanisms that CRC cells exploit to transition from a sensitive to a tolerant state could disrupt their intrinsic or adaptive resistance, thereby improving therapeutic outcomes. Expanding on this rationale, our study compels evidence for a non-genetic molecular mechanism that entails chromatin conformational alterations induced quiescence and heightened transcriptional variability involved in the survival response of CRC cells to the absence of KRAS.

## Materials and Methods

Detailed materials and methods are provided in supplementary information (Supplementary File 1).

### Cell lines

CRC cell lines HCT116 (RRID:CVCL_0291) and SW480 (RRID:CVCL_0546) were purchased from the American Type Culture Collection (ATCC). LS174 T cells were provided by Dr. Ragnhild A. Lothe (Oslo University Hospital, Norway). All cell lines were maintained under standard culture condition.

### siRNA transfection

Prior to three-dimensional (3D) culture, CRC cell lines were seeded in two-dimensional (2D) conditions in six-well plates, cultured for approximately 16 hours, and then transfected with siRNA according to the protocol described at Supplementary File 1.

Twenty-four hours post-transfection, cells were seeded in 3D CoSeedis (abc biopply, Switzerland) or microwells prepared with micro-molds (3D Petri Dish^®^, by MicroTissues, Inc., USA). Cell seeding was performed at a density of 1 × 10^5^ cells mL^−1^, corresponding to 1000 cells per microwell. 3D cultures were incubated for 48 hours, after which optical microscopy was used to assess cell growth and spheroid formation. KRAS silencing efficiency was evaluated by Western blotting (Fig. S1).

### Cell cycle and apoptosis analysis

The cell cycle was assessed using Click-iT Plus EdU Alexa Fluor 647 Flow Cytometry Assay kit (Invitrogen, ThermoFisher Scientific, USA), following the manufacturer’s instructions. Apoptosis was evaluated using annexin V-FITC and PI double staining followed by flow cytometry (BD Pharmingen^™^ FITC Annexin V Apoptosis Detection Kit), following the protocol provided by the manufacturer. The results were analyzed with FlowJo software version X and are presented as the mean ± standard deviation of three biological replicates of each condition.

### Spheroid characterization

After 48 hours of 3D culture, spheroids were imaged using the IN Cell Analyzer 2000. Spheroids’ area, diameter, circularity, and solidity were quantified using Fiji software.

### Cell lysis and protein extraction

Cells were washed twice with PBS, followed by slight dissociation with Accutase (GRiSP, Portugal) for 30 minutes at 37°C. Spheroids were recovered, pelleted and then resuspended in cell lysis buffer containing 1% IGEPAL CA-630, 1% Triton X-100 supplemented with a protease inhibitor cocktail (Roche, Switzerland) and a phosphatase inhibitor cocktail (Sigma Aldrich, USA). Cell lysates were further incubated on ice for 30 minutes and centrifuged for 20 minutes (13300 rpm, at 4°C) to pellet the insoluble material. Protein concentration was determined using a DCProtein assay kit from BioRad (USA). The extracted proteins were stored at −20 °C until further analysis.

### Sample processing for proteomics and LC-MS/MS analysis

Cells were lysed for protein extraction according to the protocol described at Supplementary File 1. One hundred micrograms of protein were eluted with elution buffer (60 mM Tris–HCl, 10% glycerol, 2% SDS, and 5% 2-mercaptoethanol, pH 6.8) at 95°C for 5 minutes. To prepare the samples for proteomic analysis, single-step reduction and alkylation with tris-2(-carboxyethyl)-phosphine (TCEP)/chloroacetamide (CAA) were performed in combination with the single-pot solid-phase-enhanced sample preparation protocol as described elsewhere [27,28].

Protein identification and quantitation were performed by nanoLC–MS/MS using an Ultimate 3000 liquid chromatography system coupled to a Q Exactive Hybrid Quadrupole-Orbitrap mass spectrometer (Thermo Fisher Scientific, Bremen, Germany).

### Bioinformatic Analysis

The acquired raw data were analyzed using the MaxQuant search engine 1.5.3.30 for protein identification and label-free quantification (LFQ) (iBAQ intensity analysis). The data were searched against the UniProt human database (UP000005640, downloaded from October 2020).

Software Perseus 1.6.14.0 was used for the data processing and statistical analysis. All the datasets were subjected to Student’s t-test, and a p-value less than 0.05 and a fold change greater than 2 were considered to indicate statistical significance.

Gene Ontology (GO) pathway analysis was performed using DAVID Bioinformatics. Network analysis was performed with the Search Tool for the Retrieval of Interacting Genes/Proteins (STRING). The acquired raw data were also analyzed using Virtual Expert Mass Spectrometrist software for the identification of posttranslational modifications (PTMs).

### Histone acid extraction

Histones were extracted by an acid extraction method (adapted from [29]). The protein concentration was determined using a DCP protein assay kit from Bio-Rad (California, USA). The extracted proteins were stored at −20 °C until further analysis.

### Western blotting

Protein expression validation was performed by western blotting. Protein lysates were separated via 4–20% sodium dodecyl sulfate‒polyacrylamide gel electrophoresis under denaturing conditions. Details on membrane blocking and primary and secondary antibody incubation and signal visualization and quantification are described at Supplementary File 1.

### Transmission electron microscopy (TEM)

For the ultrastructure analysis, cells were treated following standard TEM protocol and visualized on a JEOL JEM 1400 transmission electron microscope (JEOL, Tokyo, Japan), and the images were digitally recorded using a CCD digital camera Orius 1100W (Tokyo, Japan).

### RNA extraction and Sequencing

For the analysis of RNA expression at 72 hours after KRAS silencing, total RNA was submitted to TruSeq mRNA-Seq library preparation and sequenced in an Illumina HiSeq 4000 Sequencer using a paired-end 50-base pair strategy.

For longitudinal RNA expression analysis, libraries were constructed from purified total RNA and sequenced on an Illumina NovaSeq 6000 platform using a paired-end 150-base pair strategy.

### RNA sequencing data analysis

Reads were aligned to the human genome (hg38) using STAR and Counts and transcripts per million for each condition were estimated from mapped reads using RSEM. Subsequent analysis was performed using RStudio version 2023.06.0.

### Partial Wave Spectroscopic Microscopy

Fixed spheroid embedded in paraffin were deparaffinized for Partial Wave Spectroscopic Microscopy analysis (detailed on supp mat e met) to collect spectrally resolved images between 500 to 700 nm with 1 nm step size. At least 40 cells were quantified from each condition. Three biological replicates were used for the analysis.

### Preparation of Hi-C Libraries, Hi-C processing and analysis

Hi-C was generated using the Arima-HiC Kit, according to the manufacturer’s protocols. Library preparation was performed using Illumina primers and protocol and amplified following the manufacturer’s protocol. Paired-end sequencing was performed by Admera health using the Illumina HiSeq 2000 OR 2500 platform. Hi-C data was then processed using Juicer tool [30].

### Statistical analysis

Statistical analysis was performed using the GraphPad Prism version 9.0 (GraphPad Software, Inc., USA). Normality was tested using the Shapiro-Wilk test. Paired t-tests and two-way ANOVA or nonparametric equivalent tests were used according to the most adequate test for each comparison. The test used for each comparison is indicated in the figure legends. Significance was defined as a p-value* ≤0.05, p-value** ≤0.01, p-value*** ≤0.001, and p-value**** ≤0.0001.

## Results

### KRAS dependency is retained in spheroids from colorectal cancer cell lines

Three mutant KRAS CRC cell lines were used in this study (HCT116, SW480, and LS174T). Despite all harboring KRAS mutations, in 2D cell culture models, the three cell lines were previously shown to display distinct degrees of dependency on KRAS oncogenic signaling to survive [31]. To validate KRAS dependency in 3D cell cultures, 24 hours after KRAS inhibition, cells were seeded in 3D conditions and cultured for another 48 hours. As such, all the analyses were performed 72 hours after KRAS inhibition (Fig. S1A-B).

**Figure 1:**
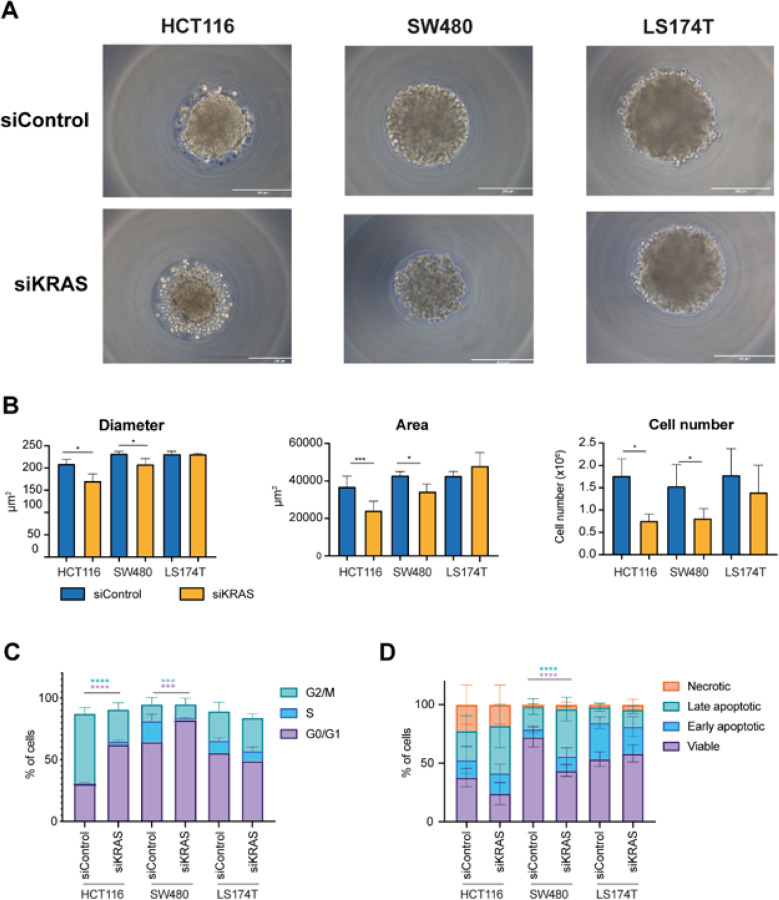
Colorectal cancer cells retain KRAS dependency in 3D cell cultures. HCT116, SW480, and LS174T cells, both siControl and siKRAS, were grown as spheroids for 48 hours. **A** Representative microscopy images of spheroids of siControl and siKRAS cells (scale bar: 200 μm). **B** Quantification of spheroids diameter, area and cell number; paired t-test was used for statistical analysis (*P≤0.05; ***P ≤0.001). **C** Cell cycle analysis: bar graphs illustrate the percentage of cells in the different cell cycle phases (G0/G1, S, G2/M); Two-way ANOVA with multiple comparisons was used to compare different populations in siControl with siKRAS conditions (*P≤0.05; **P ≤0.01; ***P ≤0.001). **D** Apoptosis analysis: bar graphs represent the percentage of cells that are in viable, early or late apoptosis, and necrotic statesTwo-way ANOVA with multiple comparisons was used to compare different populations in siControl with siKRAS conditions (*P≤0.05; **P ≤0.01; ***P ≤0.001; ****P ≤00001). All the bar graphs represent the mean ± SD of at least three independent experiments.

The three cell lines formed spheroids in less than 24 hours after seeding, either with a compact (HCT116) or loose (SW480 and LS174T) morphology (Fig. 1A). Although all the cell lines exhibited a high degree of KRAS silencing (Fig. S1A), only HCT116 and SW480 cell lines exhibited KRAS-signaling dependency. In both cell lines, spheroids formed by siKRAS cells had a smaller area and diameter compared with spheroids formed by siControl cells, along with a reduction in the number of cells (Fig. 1B). Additionally, HCT116 siKRAS spheroids tended to exhibit decreased circularity and a significant decrease in solidity (Fig. S2). No significant alterations were observed between the siControl and siKRAS LS174T spheroids (Fig. 1A–B), further corroborating the independence of KRAS signaling of this cell line.

Next, we evaluated the impact of KRAS silencing on cell cycle progression and apoptosis by flow cytometry. The gating strategy used for cell cycle and apoptosis analysis can be found as supplementary data (Fig. S3). Compared with siControl cells, siKRAS HCT116 cells exhibited a significant increase in the G0/G1 phase and a decrease in the G2/M phase. siKRAS SW480 cells exhibited a significant increase in the G0/G1 population and a decrease in the S phase population (Fig. 1C) compared with their respective siControl. Regarding apoptosis, statistically significant differences were only detected in siKRAS SW480 cells, which showed an increase in late apoptotic cells compared with siControl cells (Fig. 1D). No differences were observed for KRAS-signaling independent LS174T cells, either in terms of cell cycle or apoptosis (Fig. 1C–D).

The quiescent state of siControl and siKRAS cells was calculated using an algorithm that defines a G0 arrest score derived from transcriptomic data [32]. Independent of the cell line analyzed, siKRAS cells presented a positive score, thus indicating the induction of a G0 arrest signature (Fig. S4). Notably, the G0 arrest score of siKRAS HCT116 and SW480 cells was higher than the LS174T score.

In summary, our results confirm that SW480 CRC cells are sensitive to KRAS silencing, and LS174T cells are resistant. HCT116 cells, although originally classified as KRAS-independent based on survival analysis in 2D cell cultures, are herein classified as sensitive to KRAS silencing based on the proliferation and quiescence results.

### 3.2 CRC cells sensitive to KRAS silencing exhibit a proteomic profile indicative of chromatin and transcriptional reprogramming

**Figure 2:**
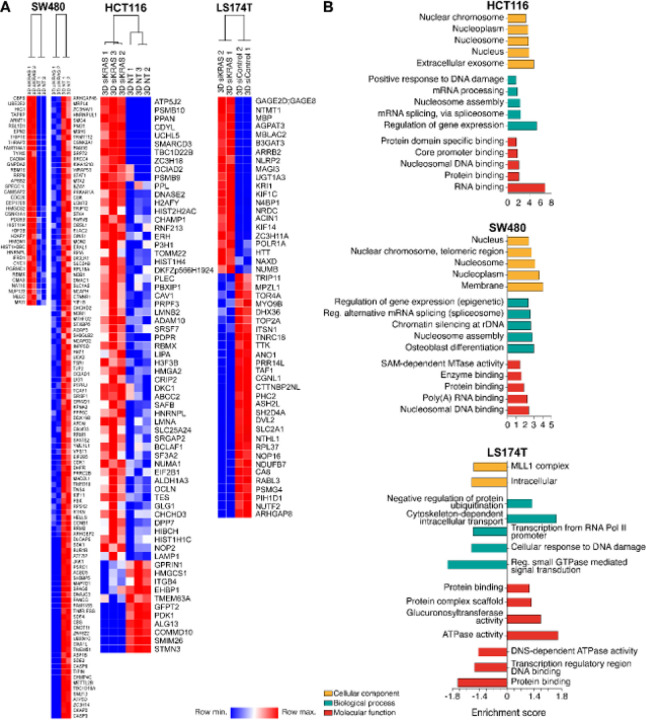
Spheroids from KRAS-dependent silenced cells exhibit a proteomic profile indicative of chromatin reprogramming. **A** Heatmap of up- and downregulated proteins. Proteomic analysis was performed in three biology replicates of HCT116 and two biologic replicates of SW480 and LS174T CRC cells. **B** Gene ontology (GO) enrichment analysis of differently expressed proteins between siKRAS and siControl cells; the up-regulated and downregulated proteins were classified according to their cellular component (yellow bars), molecular function (red bars) and biological processes (green bars); The enrichment score of GO terms was calculated by −log p-value; the graphs show the top 5 GO terms sorted by p-value.

To dissect the main cellular programs altered upon KRAS silencing, we profiled the proteome of siControl and siKRAS CRC spheroids. Principal component analysis revealed a complete discrimination between siControl and siKRAS cells in HCT116 and SW480 (Fig. S5), while in LS174T that discrimination was not observed (Fig. S5). No significant changes were observed in the number of identified proteins between siControl and siKRAS cells in all the cell lines (Fig. S5 and Table S2). A total of 55 proteins were upregulated and 11 proteins were downregulated in siKRAS HCT116 cells; 38 proteins were upregulated and 113 were downregulated in siKRAS SW480 cells; 21 proteins were upregulated and 29 were downregulated in siKRAS LS174T cells compared with the respective siControl cells (Fig. 2A and Table S2). Proteomic data was validated by western blotting for randomly selected proteins (Fig. S6).

Gene Ontology (GO) analyses revealed upregulation of proteins mainly localized at nuclear compartments (nucleus, nucleosome, and nucleoplasm), extracellular exosome, and in the membrane (Fig. 2B) in the two KRAS-silencing sensitive cell lines, HCT116 and SW480. Upregulated proteins in LS174T cells were not associated with any cellular component. For the downregulated proteins, the cellular component category was only possible to be determined in SW480 and LS174T cells. In SW480, proteins were mainly localized in cytosol and cytoplasm (Table S3), while in LS174T cells, proteins were mainly localized intracellularly and in the MLL1 complex (Fig. 2B). The biological processes category revealed that in HCT116 and SW480 cells upregulated proteins were mainly associated with regulation of gene expression, mRNA splicing, nucleosome assembly, and mRNA processing, among others, while in LS174T cells were associated with intracellular transport and regulation of protein ubiquitination. Downregulated proteins were mainly associated with neuron projection development in HCT116, cell division and cell-cell adhesion in SW480, and signal transduction, response to DNA damage, and transcription in LS174T (Table S3 and Fig. 2B). Molecular function terms for upregulated proteins were mainly associated with binding activities (RNA, protein, nucleosome, and core promoter binding) in HCT116 and SW480 cells, and associated with ATPase activity in LS174T cells. For the downregulated proteins, it was only possible to obtain information for SW480 and LS174T, and in both, they were associated with binding activities (Fig. 2B and Table S3).

STRING was used to determine possible protein-protein interactions among the proteins differentially expressed between siControl and siKRAS cells (Fig. S7). Proteins that were upregulated in HCT116 siKRAS cells formed two major clusters: one of the clusters was composed of proteins associated with the nucleosome, including HMGA2, HIST1H1C, HIST1H4F, HIST2H2AC, H3F3B and H2AFY; the other cluster was composed of mRNA splicing-related proteins, such as SF3A2, PRPF3, RBMX, SRSF7, SAFB and HNRNPL (Fig. S7A). In SW480 siKRAS cells, upregulated proteins formed a cluster associated with nucleosomes that included proteins such as H3F3B, HIST1H4F, HIST1H2BC, and H2AFY and a cluster associated with chromosome condensation formed by downregulated proteins such as SMC4, NCAPD2, CCNB1, CDK1, PBK, DLGAP5, CKAP2, SPAG5 and KIF11 (Fig. S7B). Proteins differentially expressed in LS174T siKRAS cells did not exhibit significant interactions and did not show any specific clustering (Fig. S7C).

In summary, KRAS silencing significantly impacted the cellular proteome, exhibiting distinct effects on KRAS-silencing sensitive and resistant cell lines. KRAS silencing in sensitive cells enhanced proteome remodeling within the nuclear compartment, influencing the expression of proteins associated with chromatin and the regulation of gene expression.

### 3.3 KRAS silencing impacts the physical organization of chromatin

Given that a significant number of the upregulated proteins in KRAS-silencing sensitive cell lines were localized within the nucleus (such as the nucleoplasm, nucleolus, and chromosomes) and that the affected biological processes were associated primarily with chromatin reorganization, nucleosome assembly, and gene expression, we further aimed to characterize the status of chromatin organization in siControl and siKRAS cells.

Histone post-translational modifications (PTMs) play a fundamental role as epigenetic regulators of chromatin accessibility and gene expression. Therefore, we explored our proteomic data for alterations in histone PTMs to assess whether this could be a mechanism involved in chromatin remodeling. Acetylation of lysine 18 and 23 in histone 3 (H3k18ac, H3k23ac) and acetylation of lysine 8, 12, and 16 in histone 4 (H4k8ac, H4k12ac, H4k16ac), marks associated with active transcription, were found in KRAS-silenced sensitive cell lines (Fig. S8). Through western blotting, we were not able to validate H3K18ac found in the proteomics (Fig. S9A–B).

In addition, the analysis of other histone PTMs commonly associated with regulatory elements revealed that only HCT116 siKRAS cells showed a significant decrease in H3k9ac. Besides that, no other changes in the nuclear levels of histone 3 PTMs were found in siKRAS HCT116 and siKRAS SW480 (Fig. S9A–B). The results for the LS174T cell line were inconclusive due to very weak signals despite the increased amounts of protein used (Fig. S9C).

**Figure 3:**
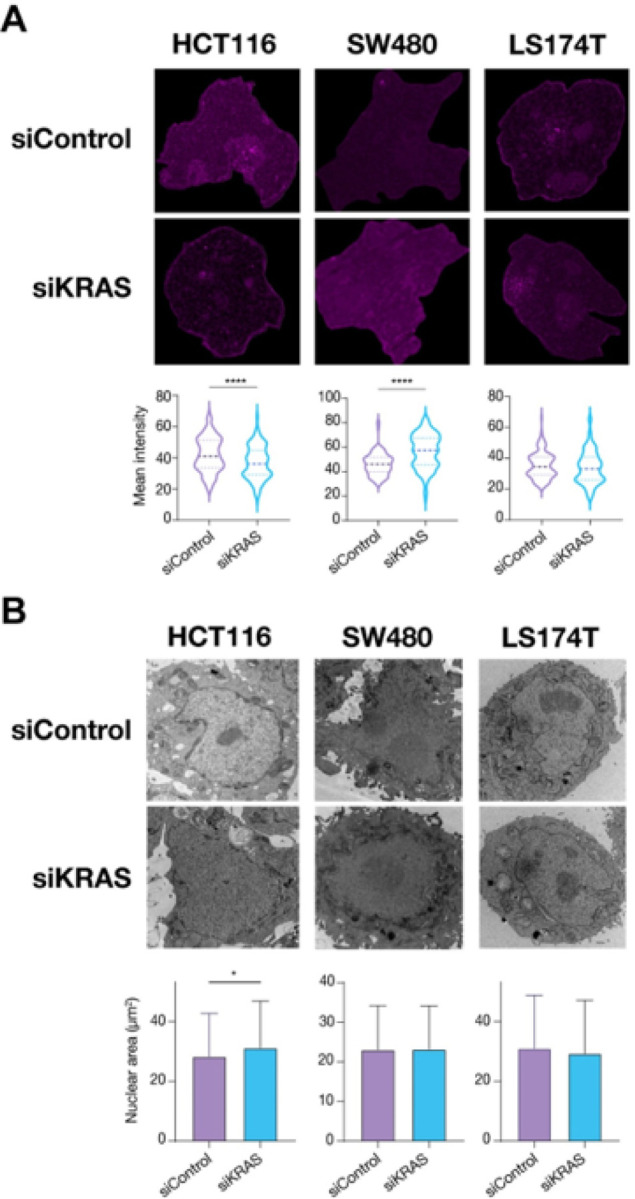
KRAS-dependent CRC cells show alterations at the level of chromatin compaction upon KRAS silencing. **A** Representative electron microscopy images showing areas of euchromatin (dark areas) and heterochromatin regions (bright purple), and respective Mean Intensity quantification of heterochromatin for each cell line. **B** Nucleus area quantified using Fiji. For quantification, three different experiments were performed and around 60 nucleus per condition were analyzed for each experiment. Data was obtained from three biological replicates for each condition. Mann-Whitney test was used to perform all analysis; *P≤0.05, **P ≤ 0.01, ***P ≤ 0.001, ****P ≤ 0.0001.

Furthermore, the global proteomic changes identified in our study suggested that KRAS silencing may affect the higher-order packing state of chromatin. Higher-order packing of chromatin is intimately linked to overall transcription regulation, though it encompasses hierarchical compartmentalization of the genome into subdomains at different genomic scales. Seventy-two hours after KRAS silencing, the two siKRAS-sensitive cell lines exhibited alterations in chromatin packing, whereas no alterations were found in the siKRAS-resistant cell line (Fig. 3A). In HCT116 siKRAS cells, the level of heterochromatin decreased concomitantly with an increase in the nuclear area; in turn, SW480 siKRAS cells displayed an increase in heterochromatin without changes in the nuclear area (Fig. 3A–B).

**Figure 4:**
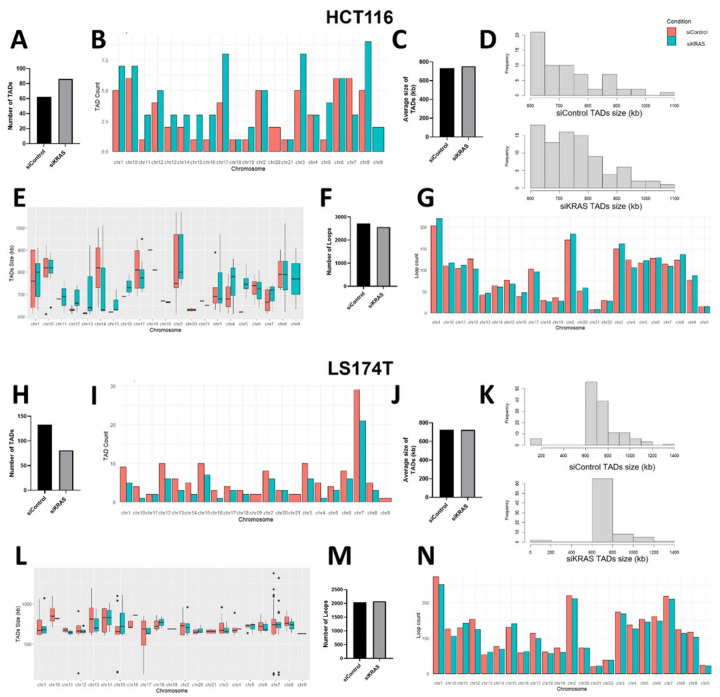
Hi-C shows alterations of genome-wide chromatin interactions upon KRAS inhibition. **A** Graph represents number of TADs identified in HCT116 cells. **B** Graph represents number of TADs identified per chromosome in siControl and siKRAS HCT116 cells. **C** Graph represents the average size of TADs identified in HCT116 cells. **D** Graphs represent frequency distribution of TADs size in siControl and siKRAS HCT116 cells. **E**Graph represents size of TADs identified in each chromosome in HCT116 cells.F Graph represents number of loops identified in HCT116 cells. **G**Graph represents number of loops identified per chromosome in siControl and siKRAS HCT116 cells. **H** Graph represents number of TADs identified in LS174T cells. **I** Graph represents number of TADs identified per chromosome in siControl and siKRAS LS174T cells. **J** Graph represents the average size of TADs identified in LS174T cells. **K** Graphs represent frequency distribution of TADs size in siControl and siKRAS LS174T cells. **L**Graph represents size of TADs identified in each chromosome in LS1774T cells. **M** Graph represents number of loops identified in LS174T cells. **N** Graph represents number of loops identified per chromosome in siControl and siKRAS LS174T cells.

At a supra nucleosomal scale, chromatin organizes into topologically associating domains (TADs), thus demarcating an environment for preferred interactions [33]. Loops are formed within TADs to mediate specific enhancer-promoter interactions [34]. Genome-wide DNA interactions induced by KRAS silencing were mapped in one siKRAS-sensitive (HCT116) and one resistance (LS174T) cell line through high-throughput chromosome conformation capture sequencing (Hi-C). In the HCT116 cell line, more than 60 million contacts were generated for siControl (64,458,010) and siKRAS cells (70,658,657). In LS174T, 70,904,760 million contacts were generated for siControl and 60,276,286 contacts for siKRAS cells. Using arrowhead analysis, 62 TADs were identified on siControl HCT116 cells, while 86 TADs were found on siKRAS HCT116 cells (Fig. 4A–B). The average size of TADs did not change from siControl (~735kb) to siKRAS (~754kb) HCT116 cells (Fig. 4C). Nonetheless, changes in size frequency were observed. Specifically, siKRAS cells exhibited a higher prevalence of larger TADs compared to siControl cells (Fig. 4C–E). In LS174T cells, the number of TADs decreased from 133 found in siControl to 81 found in siKRAS cells (Fig. 4H–I). TADs average size did not change between siControl (~728kb) and siKRAS (~723kb) conditions (Fig. 4J), and no major differences regarding size frequency were observed (Fig. 4J–L).

**Figure 5:**
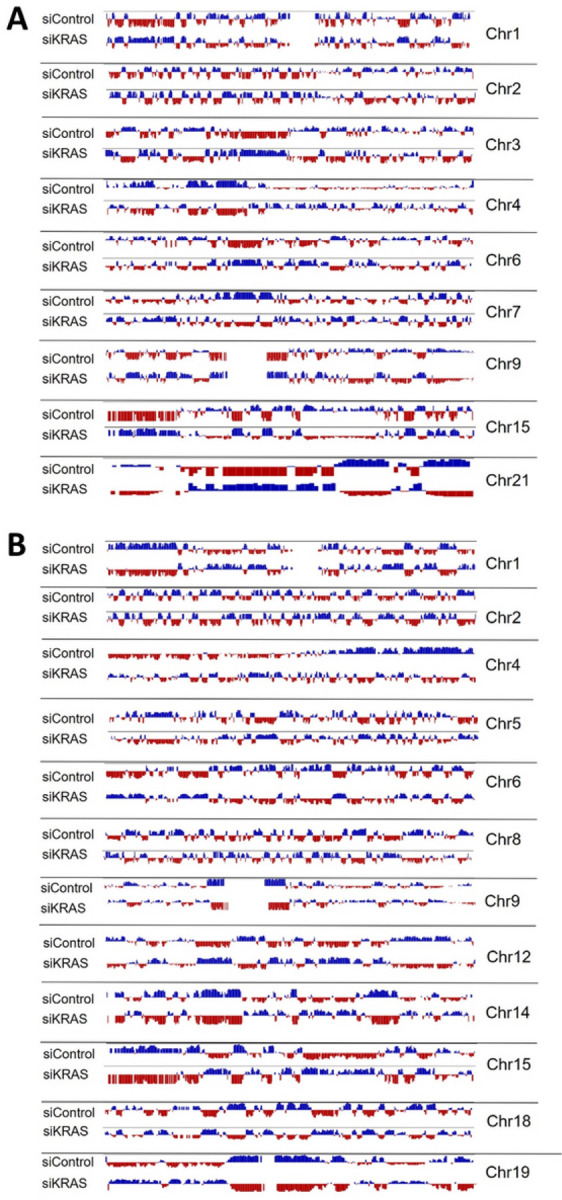
Hi-C shows alterations of A/B compartment upon KRAS inhibition. **A** Figure displays data of A/B compartment distribution in several chromosomes in siControl and siKRAS conditions for HCT116 cells. **B** Figure displays data of A/B compartment distribution in several chromosomes in siControl and siKRAS conditions for LS174T cells. Data was obtained through eigenvector.

No correlation was found between changes in TADs number and differential transcriptional profile of siKRAS HCT116 and LS174T (Table S4). In addition to TADs, we also evaluated the number of chromatin loops. More than 2000 loops were identified in both cell lines in siControl or siKRAS conditions. Nonetheless, there were no major alterations in the total number of loops found between siControl or siKRAS (Fig. 4F and Fig. 4M), nor in the number of loops found per chromosome (Fig. 4G and Fig. 4N) in the two cell lines. Moreover, the Hi-C long-range contact frequency matrix also provides a distribution of the chromatin into A and B compartments, corresponding to transcriptionally active and repressive regions, respectively [35]. In the two cell lines, no alterations were found in the global levels of chromatin within each compartment between siControl and siKRAS conditions (Fig. S10). However, systematic analysis of A/B compartment tracks per chromosome revealed regions in several chromosomes where the compartment identity shifted (Fig. 5) in the siKRAS condition compared to the siControl in both cell lines.

**Figure 6:**
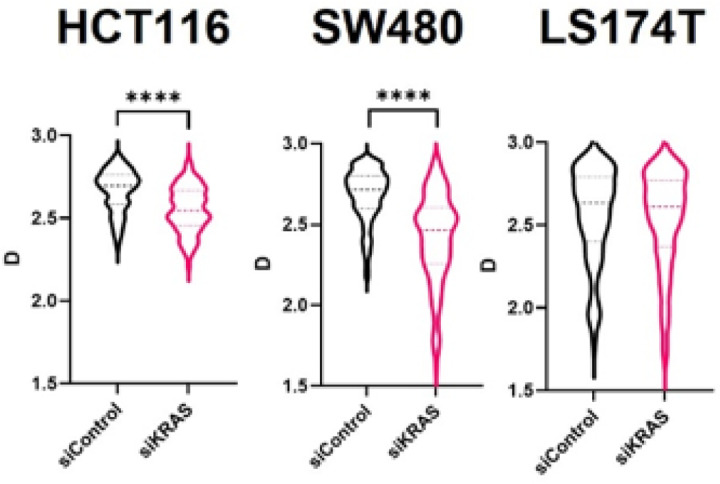
KRAS inhibition impacts chromatin packing scaling. Violin plots showing chromatin packing scaling (D) in siControl and siKRAS conditions of each cell line. The violin plots extend from the minimum to the maximum value. The line in the middle of each plot is the median value of the distribution, and the lines above and below are the third and first quartiles, respectively. Data was obtained from three biological replicates for each condition. Mann-Whitney test was used to perform all analysis; *P≤0.05, **P ≤0.01, ***P ≤0.001, ****P ≤0.0001.

Inside the nucleus, chromatin organizes into several thousand packing domains of variable sizes, densities, and fractal-like internal conformation, which influence the accessibility of chromatin to transcription regulators [36,37]. The capacity of cells to form new packing domains and the chromatin conformation within and outside of packing domains within the nucleus was recently demonstrated to have a strong influence on global gene expression patterns and the adaptation and survival of cancer cells to chemotherapy. This phenomenon can be quantified by the average nuclear chromatin domain packing scaling. Specifically, higher chromatin packing scaling corresponds to packing domain upregulation and/or increased fractality of their internal conformation and heterogeneity, thus engendering higher transcriptional malleability, heterogeneity, and plasticity [37,38]. Conversely, packing domain downregulation is indicative of a shift of chromatin structure towards less plastic and dormant states [39]. In order to evaluate whether cells respond to KRAS silencing by changing the 3D physical organization of their genome within the nucleus, we performed PWS to characterize the chromatin packing scaling on fixed spheroids. Our results showed a decrease in the packing scaling of chromatin upon KRAS silencing in the two siKRAS-sensitive cell lines, HCT116 and SW480 cell lines, whereas no changes were found in siKRAS-resistant LS174T cells when compared with the respective siCTRL cells (Fig. 6).

Altogether, these observations support chromatin structural changes as part of the KRAS-dependent CRC cell response to KRAS silencing.

### 3.4 KRAS silencing enhances transcriptional dynamics in sensitive cell lines

**Figure 7:**
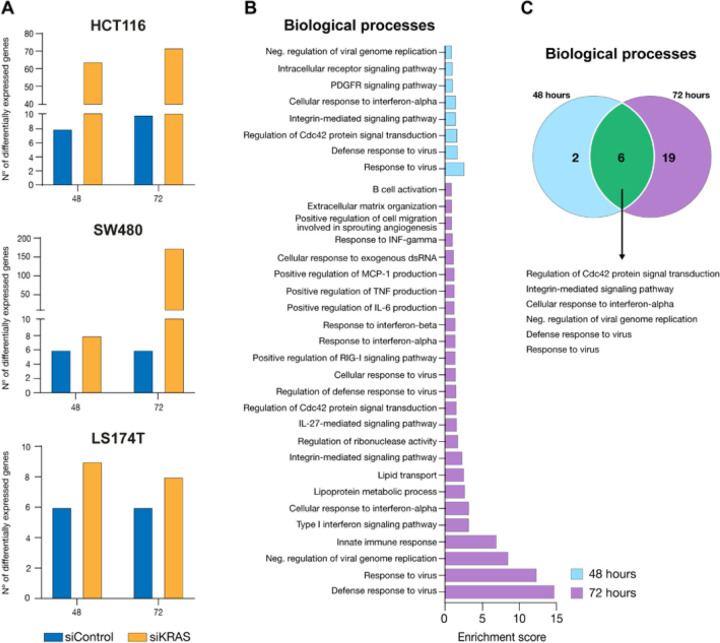
KRAS-dependent CRC cells exhibit higher transcriptional dynamics upon KRAS silencing. A Number of differentially expressed genes in siControl and siKRAS at the different time-points, compared with their respective 24-hour timepoint. B GO terms (biological processes) of differentially expressed genes between 48- and 72-hour and the 24-hour timepoint in siKRAS from HCT116 cells. The enrichment score was calculated by −log p-value. C Venn diagram illustrating the number of unique and shared biological processes altered at 48- and 72-hour timepoints in siKRAS HCT116 cells. Data is representative of one experiment. Differentially expressed genes were obtained through NOISeq R package, a tool that allows differential expression analysis from RNA-seq data with no replicates.

Recognizing the significance of transcriptional plasticity and heterogeneity in ensuring the survival and adaptation of cancer cells to stress [38,40] and the reported impact that chromatin packing domains have on both [36,38], we then evaluated how variable the transcriptomic profile within siControl and siKRAS cells was. To do so, we performed RNA-seq at distinct timepoints (12-, 24-, 48-, and 72-hour) upon siRNA transfection in the two siKRAS-sensitive cell lines and the one siKRAS-resistant. Although KRAS mRNA expression was highly reduced at 12 hours post-siRNA transfection (Fig. S11A), protein downregulation was observed only at the 24-hour timepoint, and it was maintained throughout the following timepoints (Fig. S11B-C). As such, we considered the 24-hour timepoint as the basal condition against which the subsequent timepoints (24- and 48-hour) were compared. This approach ensured that we did not compare conditions with highly disparate levels of KRAS protein. No significant differences were observed in the total counts between the different timepoints or between the siControl and siKRAS cells (Fig. S12A). A multidimensional scaling plot (Fig. S12B) revealed that siKRAS differ more between themselves over time than siControl cells. By comparing the number of DEGs in each condition (siControl or siKRAS) at the 48- and 72-hour timepoints with that from the 24-hour timepoint (the first timepoint at which the maximum KRAS protein inhibition was reached), siKRAS-sensitive cell lines were found to be transcriptionally more dynamic than siControl cells were, as evidenced by a greater number of DEGs over time (Fig. 7A). Specifically, in HCT116 siKRAS cells, 64 and 72 genes were differentially expressed between the 24-hour timepoint and the 48- and 72-hour timepoints, respectively, whereas only 8 and 10 genes were differentially expressed at the same timepoints in siControl cells (Fig. 7A, Table S5). Additionally, only half of the DEGs were shared between the 48- and 72-hour timepoints (Fig. S13A and Table S6), thus suggesting that transcriptional changes were not merely incremental but rather dynamic, with half of the genes being differentially regulated.

Compared with siControl cells, SW480 siKRAS cells also demonstrated greater transcriptional variation, especially at 72 hours (Fig. 7A). While only 8 genes were differentially expressed at the 48-hour timepoint compared to the 24-hour timepoint, 174 genes were differentially expressed at 72 hours. In the siKRAS-resistant cell line LS174T, the variations in the transcriptome of siKRAS cells was very similar to that of siControl cells, with very few genes exhibiting changes in expression between timepoints (Fig. 7A). We then inquired whether the variability in gene expression across timepoints would lead to similar functional outcomes or alter the functional repertoire of the tolerant cells. This analysis was exclusively performed on HCT116 siKRAS cells due to their sufficiently high number of DEGs, which enabled the analysis at both timepoints. At 48 hours, eight biological processes were upregulated (Fig. 7B and Table S5). At 72 hours, a total of 25 biological processes were upregulated: six were shared with the 48-hour timepoint, and 19 were acquired *de novo* (Fig. 7B–C). Shared biological processes were mainly related to response to virus and regulation of CDC42 signaling. Within the acquired biological processes, we still observed a gain in processes related to response to virus, alongside processes associated with immune regulation, extracellular matrix remodeling, cell motility associated with angiogenesis, and lipid metabolism and transport. Therefore, we conclude that the enhanced transcriptional dynamics observed in HCT116 siKRAS cells not only preserve certain functional outcomes but also introduce a diverse array of new functional possibilities.

In summary, the analysis of transcriptional activity revealed that siKRAS-sensitive cells exhibited a marked increase in transcriptional dynamics, resulting in heightened diversification of biological processes across time. This emergent functional diversity may play a crucial role in the selection of resistant clones.

## Discussion

Cancer cells exhibit a remarkable capacity for adaptive resistance to treatments, particularly in the context of targeted inhibition of the oncogenic signal they rely upon [41]. The clinical observations of enhanced tolerance to KRAS inhibition in CRC clearly typify this paradox. Although KRAS oncogenic activation is a well-established driver of CRC initiation and progression, its inhibition has not translated into clinical efficacy. Clinical data has revealed that the most common outcome in patients treated with KRAS inhibitors is stable disease [42], suggesting that while initially sensitive to treatment, they rapidly develop tolerance. In our study, we took advantage of 3D *in vitro* models to shed light on the mechanisms underlying the switch in sensitivity. Our findings demonstrate the remodeling of 3D chromatin structure as a potential epigenetic mechanism enabling cancer cells to tolerate and withstand KRAS inhibition.

To better discern the mechanisms of tolerance to treatment, we used CRC cell lines with distinct sensitivities to KRAS silencing. KRAS-silenced cells from the two KRAS-dependent cell lines were arrested in the G0/G1 phase of the cell cycle. This observation is consistent with the findings of preclinical and clinical studies, which showed that G0/G1 cell cycle arrest is a common feature of cancer cells after treatment with different KRAS inhibitors, including G12C, G12D, and pan-RAS inhibitors [43–45]. Moreover, we also identified a transcriptomic signature of quiescence after KRAS silencing, which has previously been linked to therapeutic resistance [32]. A timely transition to a quiescent-like, slow-cycling state, often associated with reversible G0/G1 cell cycle arrest, is a typical strategy applied by proliferative cancer cells to evade cell death when exposed to stress conditions. For example, metastatic cancer cells can remain dormant for long periods of time while adapting to new environments, and drug-sensitive proliferative cells switch to a drug-tolerant, slow-cycling state, a critical predecessor of therapy-resistant states [32,44,46]. Thus, despite diminishing tumor growth, cell cycle arrest and quiescence allow KRAS-dependent CRC cells to survive KRAS silencing.

In order to characterize the molecular transformations undergone in KRAS-silenced quiescent cells that may be accountable for their transition into a non-proliferative state, we profiled the proteome of cells that survived KRAS silencing, highlighting chromatin remodeling and regulation of transcription-associated processes as the main biological processes up-regulated in siKRAS-sensitive cells. These observations were validated by subsequent studies detailing the type of alterations at the chromatin and transcriptional level. Specifically, chromatin conformational studies revealed alterations in the compaction and topological organization. Global nuclear analysis of chromatin compaction revealed that only siKRAS-sensitive cell lines changed their heterochromatin levels, whereas no changes were found in the siKRAS-resistant cell line. A more in-depth analysis through Hi-C, although revealing frequent shifts in A/B compartments in siKRAS-sensitive and resistant cell lines after KRAS silencing, showed a distinct trend regarding TADs formation: while the siKRAS-resistant cell line lost TADs, the sensitive cell line gained TADs which also tended to be larger. However, the comparison between the transcriptomic profile of siKRAS and siControl cells revealed that these changes in TADs were not yet contributing much to the differences in the transcriptional repertoire. Still, given the short time window of our analysis, we cannot exclude that a gain in TADs may impact long-term adaptations of sensitive cells to KRAS silencing by expanding the array of possible enhancer-promoter interactions and, thus, their responsiveness to other stimuli. Our study also demonstrated that siKRAS-sensitive, but not siKRAS-resistant cells adjusted the 3D physical organization of their genome within the nucleus after KRAS silencing by decreasing its packing scaling. This observation contrasts with the reported increase in chromatin packing scaling upon chemotherapy treatment [38,47]. In this previous study, it was demonstrated that increased chromatin packing scaling influenced transcription malleability towards increasing the capacity of cancer cells to up-regulate initially overexpressed genes and suppress initially underexpressed genes. As such, through increasing chromatin packing scaling, cancer cells strengthen pre-existing pro-tumorigenic programs that allow them to survive the deleterious effect of chemotherapy. However, KRAS silencing, by itself, leads to a down-regulation of survival and proliferative signals that have been triggered by its oncogenic activation. As cells are no longer actively proliferating, chromatin re-organizes towards decreasing the packing scaling to allow cells to find new transcriptional programs that allow them to survive and “de novo” reprogram their genome to resume growth independently of KRAS. This assumption is corroborated by previous data associating a lower chromatin packing scaling with quiescent states ()and by our longitudinal RNA-seq data demonstrating that siKRAS-sensitive cells had a higher transcriptional dynamics across timepoints than siControl cells.

**Figure 8:**
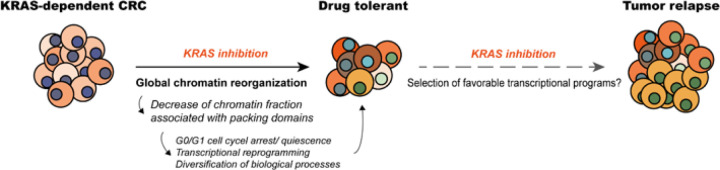
Schematic model illustrating the mechanism of tolerance to KRAS loss. Mutant KRAS fuels KRAS-dependent cancer cells with strong pro-tumorigenic signals, which shape the global fraction of chromatin associated with packing domains to support the specific transcriptional requirements. Consequently, the loss of KRAS oncogenic signaling triggers a global restructuring of chromatin, characterized by a decrease in the fraction of chromatin associated with packing domains. Cells that survive become quiescent as they lack a transcriptional backbone that allows them to thrive without the influence of mutant KRAS. Nonetheless, they exhibit a more variable transcriptional profile, hinting at a window of opportunity to undergo transcriptional reprogramming, searching for and eventually adopting new programs that enable them to circumvent KRAS dependency, thereby resuming growth.

Therefore, we propose a model through which siKRAS-sensitive cells rapidly adapt to the stress caused by KRAS inhibition, which involves decreasing the fraction of chromatin associated with packing domains that predispose cells to enter a quiescent state, concomitantly with transcriptional reprogramming that results in a diversification of functional processes (Fig.8). The observation that the siKRAS-resistant cell line, to whom KRAS inhibition does not pose any stress, maintains the original chromatin packing scaling and shows lower transcriptional dynamics supports our assumption. Long-term studies complemented with single-cell analysis and lineage tracing will be pivotal for revealing how chromatin domain structure and transcriptional dynamics co-evolve over time and impact the emergence and diversity of resistant clones. Additionally, pharmacological targeting of cancer cells’ ability to regulate chromatin domains was previously demonstrated to be highly effective in enhancing the chemotherapy effects [47]. Upcoming research will explore the potential benefits of combining KRAS inhibitors with agents targeting chromatin remodeling to enhance the overall efficacy of KRAS inhibition.

## Data Availability

RNA-Seq data discussed in this publication have been deposited in NCBI’s Gene Expression Omnibus (GEO) and are accessible through GEO Series accession number GSE249954, GSE254832 and GSE254833. The mass spectrometry proteomics data have been deposited to the ProteomeXchange Consortium via the PRIDE partner repository with the dataset identifier PXD047770. The remaining data generated in this work is contained within the article or supplementary material.

## Highlights

- KRAS silencing induces global chromatin reorganization.
- Tolerant cells survive in a quiescent, G0/G1 cell cycle arrested state.
- Chromatin-reprogrammed tolerant cells accumulate transcriptional alterations.
- Transcriptional alterations lead to a diversification of biological processes.

## Abbreviations

2D: Two-dimensional
3D: Three-dimensional
ACN: Acetonitrile
BCP: 1-Bromo-3-chloropropane
BSA: Bovine Serum Albumin
BWA: Burrows-Wheeler single end aligner
CAA: Chloroacetamide
CRC: Colorectal Cancer
DEG: Differentially Expressed Genes
EdU: 5-ethynyl-2′-deoxyuridine
EGFR: Epidermal Growth Factor Receptor
EtOH: Ethanol
FA: Formic Acid
FBS: Fetal Bovine Serum
FDR: False Discovery Rate
FOLFOX: Folinic Acid, Fluorouracil, and Oxaliplatin
GO: Gene Ontology
Hi-C: High-throughput chromosome conformation capture sequencing
ON: Overnight
PI: Propidium Iodide
PS: Penicillin–Streptomycin
PTM: Post-Translational Modifications
PWS: Partial wave spectroscopic
RNA-seq: RNA-sequencing
RT: Room temperature
SDC: Sodium deoxycholate
siControl: Control Cells
siKRAS: KRAS Silenced Cells
STRING: Search Tool for the Retrieval of Interacting Genes
TADs: Topologically associated domains
TCEP: Tris-2(-carboxyethyl)-phosphine
TEAB: Triethylammoniumbicarbonate
TEB: Triton Extraction Buffer
TEM: Transmission Electron Microscopy
