# Supplementary material for: KRAS silencing impacts chromatin organization and transcriptional activity in colorectal cancer cells
Source: Res Sq. 2024 Feb 14:rs.3.rs-3752760. Preprint. [Version 2] doi: 10.21203/rs.3.rs-3752760/v2 (PMC10896403; doi:10.21203/rs.3.rs-3752760/v2)
Supplement: Supplementary file 1 [file NIHPPrs3752760v2-supplement-1.pdf]

Supplementary Files

This is a list of supplementary files associated with this preprint. Click to download.

- SupplementaryFigures.pptx
- TableS1westernblotantibodies.pdf
- TableS2Allcelllinesallidentifiedproteinssignificantproteins.xlsx
- TableS3Functionalanalysis.xlsx
- TableS4DEG72h.xlsx
- TableS4DEG72h.xlsx
- TableS6Genesexclusivecommontimepoints.xlsx
